# Duloxetine for rehabilitation after total knee arthroplasty: a systematic review and meta-analysis

**DOI:** 10.1097/JS9.0000000000000230

**Published:** 2023-03-15

**Authors:** Jia-Man Yang, Yi Wang, Jun-Yi Li, Cong-Cong Li, Zhen-Tao Wang, Zhen Shen, Liang Ou, Ze-Hua Chen

**Affiliations:** a The Fifth Clinical College of Guangzhou University of Chinese Medicine, Guangzhou; b The orthopedics hospital of traditional Chinese medicine Zhuzhou city, Hunan; c Kunming Municipal Hospital of Traditional Chinese Medicine, Kunming; d Hunan Academy of Chinese Medicine, Changsha, China

**Keywords:** duloxetine, meta-analysis, rehabilitation, total knee arthroplasty

## Abstract

The aim was to evaluate the efficacy and safety of duloxetine for postoperative recovery after total knee arthroplasty.

**Methods:**

The following electronic databases were searched for eligible trials: PubMed, EMBASE, Web of Science, Cochrane Library, VIP, Wanfang Data, and China National Knowledge Infrastructure (CNKI). The search was performed from the inception dates to 10 August 2022. Data extraction and quality assessment were performed by two independent reviewers. Standard mean differences or mean differences with 95% CIs for pooled data were calculated. The primary outcomes were pain, physical function, and analgesic consumption. Secondary outcomes included range of motion (ROM) of the knee, depression, and mental health.

**Results:**

This meta-analysis included 11 studies, reporting on a total of 1019 patients. Results of analyses indicated that duloxetine showed a statistically significant reduction in pain at rest at 3 days, 1 week, 2, and 6 weeks and pain on movement at 5 days, 1 week, 2, 4, 6, and 8 weeks. However, there was no statistical significance in pain at rest and on movement at 24 h, 12 weeks, 6 months, and 12 months. Additionally, duloxetine had a significant improvement in physical function, ROM of the knee at 6 weeks, and emotional function (depression and mental health). Moreover, the cumulative opioid consumption at 24 h in the duloxetine groups was lower than in the control groups. But there was no statistical significance for the cumulative opioid consumption over 7 days between the duloxetine groups and controls.

**Conclusions:**

In conclusion, duloxetine might reduce pain mainly over a time span of 3 days–8 weeks and lower cumulative opioid consumption within 24 h. In addition, it improved physical function, ROM of the knee with a time span of 1–6 weeks and emotional function (depression and mental health).

## Introduction

HIGHLIGHTS

- This is the first systematic review and meta-analysis of duloxetine for postoperative recovery after total knee arthroplasty.
- The studies included in this meta-analysis are designed to be randomized controlled trials.
- Duloxetine might reduce pain mainly within postoperative 6 weeks and lower cumulative opioid consumption within 24 h after TKA.
- Duloxetine improved physical function, range of motion of the knee within 6 weeks, and emotional function (depression and mental health) after TKA.

Total knee arthroplasty (TKA) is one of the most common surgical procedures worldwide^1^. It is used for patients with end-stage knee osteoarthritis and rheumatoid arthritis^2^,^3^. There are over 700 thousand TKAs every year in the USA^4^, ~95 thousand in Australia^5^, and 17 thousand in Germany, and the incidence rate of TKA is expected to increase^6^. Although TKA has a high degree of patient satisfaction, around 20% of patients remain dissatisfied following TKA^7^. Reasons for postoperative dissatisfaction were mainly continual postoperative pain, functional disability, limited knee flexion, and pain-related mental problems^8^–^10^, which could delay postoperative recovery and rehabilitation. Therefore, the management of complications after TKA, especially to relieve continual postoperative pain, is a very important issue to recognize.

Multimodal pain management has been used to relieve postoperative pain^9^,^11^–^15^. Recently, duloxetine has been widely used in the surgical field^16^. Duloxetine is a selective norepinephrine reuptake inhibitor, which is traditionally used for its antidepressant qualities and has also been shown to be efficacious in postoperative pain^17^. Published meta-analyses have demonstrated that duloxetine could reduce immediate postoperative pain, opioid consumption, and nausea and supported the idea that duloxetine could be considered an adjuvant therapy to a multimodal analgesia regimen in orthopedic surgery^18^,^19^. Moreover, some evidence reported that duloxetine could alleviate postoperative pain and decrease morphine requirements, as well as improving physical function^20^–^22^. On the contrary, some evidence found there were no positive effects on pain and function for patients after TKA^23^–^25^. There is no consistent clinical data on duloxetine for pain relief and functional recovery after TKA. In addition, its effects on pain relief and postoperative recovery in the short-term and long-term after TKA remain unknown, and no meta-analysis has assessed. Therefore, we undertook a systematic review and meta-analysis of randomized controlled trials (RCTs) to evaluate the efficacy and safety of duloxetine for postoperative recovery after TKA.

## Methods

This is a systematic review and meta-analysis of duloxetine for rehabilitation after TKA. The work has been reported in line with PRISMA (Preferred Reporting Items for Systematic Reviews and Meta-Analyses), Supplemental Digital Content 1, http://links.lww.com/JS9/A134; Supplemental Digital Content 2, http://links.lww.com/JS9/A135; and AMSTAR, Supplemental Digital Content 3, http://links.lww.com/JS9/A136 (Assessing the methodological quality of systematic reviews) Guidelines^26^. The protocol was registered with PROSPERO (CRD42022352003).

### Data sources

The following electronic databases were searched for eligible trials: PubMed (English), EMBASE (English), Web of Science (English), Cochrane Library (English), VIP (Chinese), Wanfang Data (Chinese), and China National Knowledge Infrastructure (CNKI) (Chinese). The search was performed from the inception dates to August 10, 2022. We searched the relevant databases, including four English and three Chinese electronic databases, to maximize the search for relevant articles as comprehensively as possible. We also searched Google Scholar as a supplement. Moreover, the reference lists of all retrieved literature and relevant reviews were manually screened for potentially eligible trials. We used a combination of MeSH terms and entry words, including ‘duloxetine’, ‘duloxetine hydrochloride’, ‘total knee arthroplasty’, and ‘total knee replacement’. The details of the search strategy for different databases appeared in Supplementary Table 1, Supplemental Digital Content 4, http://links.lww.com/JS9/A137.

### Eligibility criteria

Trials were selected based on the following inclusion criteria: (1) patients undergoing TKA for primary knee osteoarthritis; (2) RCTs comparing duloxetine with placebo, no duloxetine, or nonduloxetine intervention; and (3) a full text of the trials was required. Exclusion criteria were: (1) patients who had an infection history or knee joint operation history in the past year; (2) patients who had known abnormal liver and renal function, an allergy to duloxetine, or a psychiatric disorder; (3) trials without relevant outcomes; (4) trials without a control group; (5) systematic reviews or not human studies.

### Quality assessment

The methodological quality of included studies was assessed by two reviewers (J.-M.Y. and Y.W.) based on the Cochrane Handbook for Systematic Reviews of Interventions^27^. Each study was evaluated on seven items: random sequence generation, allocation concealment, blinding of participants and personnel, blinding of outcome assessment, incomplete outcome data, selective reporting, and other bias. Each item was assessed as having a low, high, or unclear risk of bias. Disagreements were resolved by a discussion with a third reviewer (J.-Y. L.).

### Data extraction

Two reviewers (J.-M.Y and Y.W.) independently extracted the following data from the included studies: lead author; country of origin; publication year; sample size; participant characteristics; type of intervention; treatment characteristics; and study outcomes. If necessary, the corresponding authors would be contacted for additional information.

### Outcome measures

The primary outcomes were pain, physical function, and analgesic consumption. Secondary outcomes included range of motion (ROM) of the knee, depression, and mental health.

### Statistical analysis

Meta-analyses were performed using Revman (version 5.3, Cochrane Collaboration), and a *P* less than 0.05 was considered statistically significant. Data from pain and physical function outcomes were pooled and analyzed as standard mean differences (SMDs) with 95% CIs. Data from analgesic consumption, ROM of the knee, depression, and mental health outcomes were pooled and analyzed as mean differences (MDs) with 95% CIs. Heterogeneity was evaluated by Higgins *I* ^2^ statistic, which ranges from 0 to 100%. *I* ^2^ describes the percentage of the variability in effect estimates that is due to heterogeneity rather than sampling error (chance). An *I* ^2^ greater than 50%  indicates substantial heterogeneity^27^. The random-effects model was applied as we have identified clinical and methodological heterogeneity among studies^28^. Subgroup analyses were performed to identify potential determinants of efficacy. A sensitivity analysis was also conducted to explore potential sources of heterogeneity between studies. Additionally, Begg’s test and Egger’s test were used to evaluate publication bias.

## Results

### Search results

A total of 121 potentially eligible records were retrieved, and none were included via other sources. After removing duplicates, the titles and abstracts of 57 records were screened, and 26 were excluded. Full texts of 31 records were evaluated, and finally, 11 RCTs met the inclusion criteria^20^–^25^,^29^–^33^ (Supplementary Fig. 1, Supplemental Digital Content 4, http://links.lww.com/JS9/A137). Moreover, all authors checked the excluded literature to ensure that the reasons for exclusion were sufficient and objective. The reasons for excluding trials are listed in Supplementary Table 2, Supplemental Digital Content 4, http://links.lww.com/JS9/A137.

### Study characteristics

In total, 11 RCTs involving 1019 patients undergoing TKA for primary knee osteoarthritis were examined, with sample sizes ranging from 16 to 121. The mean age ranged mostly from 64 to 72 years. On average, patients were more likely to be women than men. Two studies were from USA^22^,^25^, three studies were from Korea^21^,^23^,^29^, four studies were from China^30^–^33^, one study was from Singapore,^20^ and one study was from the Netherlands^24^. For comparison of interventions, four studies compared duloxetine with placebo^20^,^22^,^25^,^30^; two studies compared duloxetine with opioid^23^,^29^; two studies compared duloxetine with celecoxib^32^,^33^; and no intervention was used in the control group among the remaining studies^21^,^24^. For the duloxetine scheme, 60 mg/day was used in three studies^20^,^22^,^25^, 40 mg/day in one study^32^, and 30 mg/day in three studies^21^,^23^,^29^ without dose decrement and increment. The total intervention period was 6 weeks in three studies^21^,^23^,^32^, 10 weeks in two studies^24^,^29^, 8 weeks in three studies^30^,^31^,^33^, and 2 weeks in two studies^22^,^25^. All included studies had a dropout rate of less than 6%. The characteristics of the included studies were summarized in Table 1.

**Table 1:** Characteristics of the included studies.

References	Country	Number of patients	Women [*N* (%)]	Age, years^a^	Type of intervention	Duloxetine scheme	Outcome	Dropout rate [*N* (%)]
Ho *et al.* 20	Singapore	TG: 23 CG: 24	TG: 17 (73.9) CG: 16 (66.7)	TG: 65.2 (7.5) CG: 65.7 (7.0)	TG: Duloxetine CG: Placebo	Oral 60 mg 2 h before surgery and on the morning of the first postoperative day	Morphine requirements at 24 and 48 h, NRS at 0.5, 1, 2, 6, 12, 24 and 48 h	None
Kim *et al.* 23,29	Korea	TG: 118 CG: 121	TG: 98 (83.1) CG: 105 (86.8)	TG: 70.0 (7.0) CG: 71.3 (7.2)	TG: Duloxetine CG: Opioid	Oral 30 mg for six weeks at the time of discharge	VAS at 6 weeks, 3 and 6 months, and 1 year, function, ROM, PCA consumption	TG: 1 (0.8) CG: 2 (1.6)
Kim *et al.* 23,29	Korea	TG: 19 CG: 20	TG: 17 (89.5) CG: 16 (80.0	TG: 71.2 (6.5) CG: 67.0 (7.1)	TG: Duloxetine CG: Opioid	Oral 30 mg/day from 2 weeks before surgery to 8 weeks after surgery	VAS at 24 h, 3, 5 days, 1, 2, 6 and 12 weeks, PCA consumption, ROM	TG: 1 (5) CG: 0 (0)
Koh *et al.* 21	Korea	TG: 40 CG: 40	TG: 35 (88.0) CG: 34 (85.0)	TG: 69.1 (5.8) CG: 68.6 (9.5)	TG: Duloxetine CG: None	Oral 30 mg 1 day before surgery and for 6 weeks after surgery	VAS at 24 h, 3, 5 days, 1, 2, 6 and 12 weeks, mental health, function, PCA consumption, depression	None
Rienstra *et al.* 24	The Netherlands	TG: 57 CG: 54	TG: 38 (66.7) CG: 31 (57.4)	TG: 61.5 (8.1) CG: 64.0 (8.7)	TG: Duloxetine CG: None	Oral 30 mg/day for 1 weeks and 60 mg/day for 7 weeks	VAS at 6 weeks, 6 months, and 12 months	None
Liu *et al.* 31	China	TG: 25 CG: 20	TG: 19 (76.0) CG: 16 (80.0)	TG: 66.4 (8.3) CG: 66.8 (8.9)	TG: Duloxetine CG: None	Oral 60 mg/day, starting from the second day after operation for 7 weeks and 30 mg/day for 1 week	WOMAC pain at 2, 4, and 8 weeks, function, depression	None
YaDeau *et al.* 25	USA	TG: 53 CG: 53	TG: 28 (52.8) CG: 26 (49.1)	TG: 67.0 (7.4) CG: 63.0 (7.5)	TG: Duloxetine CG: Placebo	Oral 60 mg/day starting from the day of surgery and continuing 14 days postoperatively	NRS at 1, 3, 14, 18 days and 6 weeks, opioid consumption, depression	None
YaDeau *et al.* 22	USA	TG: 80 CG: 80	TG: 40 (50.0) CG: 35 (44.0)	TG: 63.0 (11.0) CG: 64.0 (7.0)	TG: Duloxetine CG: Placebo	Oral 60 mg/day starting from the day of surgery and continuing 14 days postoperatively	NRS at 1, 2, 14 days, opioid consumption, depression	None
Yuan *et al.* 30	China	TG: 50 CG: 50	TG: 30 (60.0) CG: 27 (54.0)	TG: 67.8 (10.12) CG: 66.2 (9.83)	TG: Duloxetine CG: Placebo	Oral 60 mg/day since preoperative day 2 till postoperative day 14	VAS at 24 h, 7 days, ROM, opioids consumption	None
Wang *et al.* 32	China	TG: 30 CG: 30	TG: 18 (60.0) CG: 19 (63.3)	TG: 64.8 (4.2) CG: 65.4 (3.8)	TG: Duloxetine CG: Celecoxib	Oral 40 mg/day starting from the first day after operation for 6 weeks	VAS at 1, 2, 4 and 6 weeks, function	None
Zhu^33^	China	TG: 16 CG: 16	TG: 11 (68.8) CG: 12 (75.0)	TG: 66.4 (8.3) CG: 68.8 (8.9)	TG: Duloxetine CG: Celecoxib	Oral 60 mg/day starting from the first day after operation for 1 weeks and 60 mg/day for 7 weeks	WOMAC pain at 4 and 8 weeks, mental health, depression, function	None

a Data expressed as mean (SD).

CG indicates control group; NRS, numerical rating scale; PCA, patient-controlled analgesia; ROM, range of motion; TG, treatment group; VAS, visual analog scale; WOMAC, Western Ontario and McMaster Universities Osteoarthritis Index.

### Quality assessment

**Figure 1:**
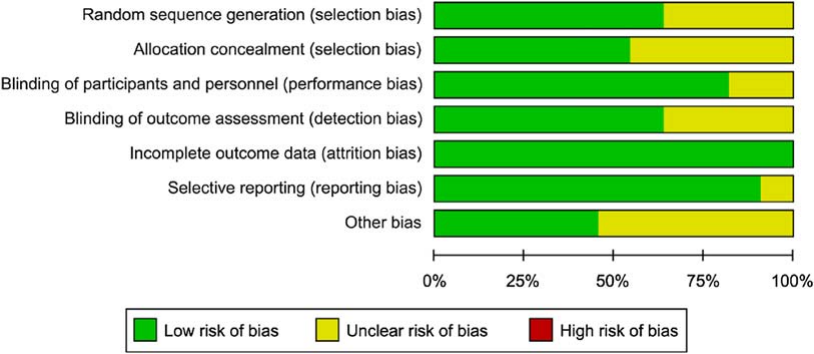
Risk of bias graph.

**Figure 2:**
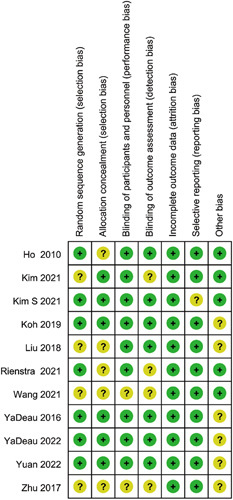
Risk of bias summary.

The methodological quality of all included studies was assessed by two reviewers (J.-M.Y. and Y.W.) according to the Cochrane Handbook for Systematic Reviews of Interventions.^27^ Seven studies^20^–^22^,^24^,^25^,^29^,^30^ reported that patients were randomly assigned to the duloxetine group or the control group according to a computer-generated randomization table. Six studies^21^–^23^,^25^,^29^,^30^ indicated that patients allocations were concealed using an opaque envelope. Nine studies^20^–^25^,^29^–^31^ described how patients and personnel were blinded to the group assignments. Seven studies^20^–^23^,^25^,^30^,^31^ reported that outcome assessment remained unaware of the patient’s allocation. All studies reported complete outcome data. Detailed results appeared in Figures 1 and 2.

### Primary outcome

#### Pain at rest

**Figure 3:**
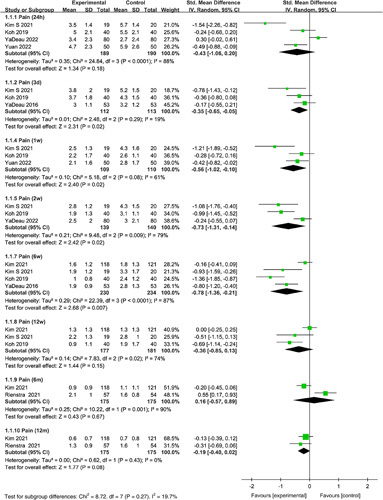
Forest plot of meta-analysis for pain at rest.

Meta-analyses were performed to evaluate the effect of duloxetine on pain at rest at 24 h, 3 days, 1 week, 2, 6, 12 weeks, 6 and 12 months after TKA. The results showed duloxetine had no statistically significant reduction in pain at rest compared with the control group at 24 h (SMD=−0.43, 95% CI: −1.06 to 0.20, *P*=0.18). Compared with the control group, duloxetine showed a statistically significant reduction in pain at rest at 3 days (SMD=−0.35, 95% CI: −0.65 to −0.05, *P*=0.02), 1 week (SMD=−0.56, 95% CI: −1.02 to −0.10, *P*=0.02), 2 (SMD=−0.73, 95% CI: −1.31 to −0.14, *P*=0.02), and 6 weeks (SMD=−0.78, 95% CI: −1.36 to −0.21, *P*=0.02). There was no statistical significance in pain at rest between the duloxetine groups and controls at 12 weeks (SMD=−0.36, 95% CI: −0.85 to 0.13, *P*=0.15), 6 months (SMD=0.16, 95% CI: −0.57 to 0.89, *P*=0.67), and 12 months (SMD=−0.19, 95% CI: −0.40 to 0.02, *P*=0.08) (Fig. 3).

#### Pain on movement

**Figure 4:**
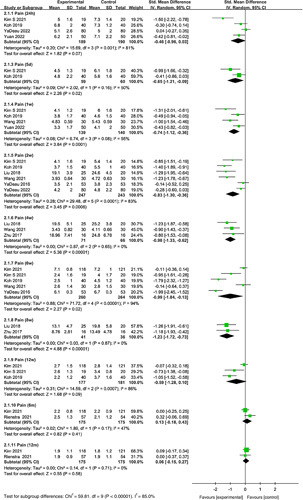
Forest plot of meta-analysis for pain on movement.

Meta-analyses were performed to evaluate the effect of duloxetine on pain and movement at 24 h, 5 days, 1 week, 2, 4, 6, 8, 12 weeks, 6 and 12 months after TKA. Pooled analysis showed duloxetine had no statistically significant reduction in pain with movement compared with the control group at 24 h (SMD=−0.46, 95% CI: −0.96 to 0.03, *P*=0.07). Compared with the control group, duloxetine showed a statistically significant reduction in pain on movement at 5 days (SMD=−0.65, 95% CI: −1.21 to −0.09, *P*=0.02), 1 week (SMD=−0.74, 95% CI: −1.12 to −0.36, *P*=0.0001), 2 (SMD=−0.83, 95% CI: −1.30 to −0.36, *P*=0.0006), 4 (SMD=−0.98, 95% CI: −1.33 to −0.62, *P*<0.00001), 6 (SMD=−0.99, 95% CI: −1.84 to −0.13, *P*=0.02), and 8 weeks (SMD=−1.23, 95% CI: −1.72 to −0.73, *P*<0.00001). There was no statistical significance in pain on movement between the duloxetine groups and controls at 12 weeks (SMD=−0.59, 95% CI: −1.28 to 0.10, *P*=0.09), 6 months (SMD=0.13, 95% CI: −0.18 to 0.43, *P*=0.41), and 12 months (SMD=0.06, 95% CI: −0.15 to 0.27, *P*=0.58) (Fig. 4).

#### Physical function

**Figure 5:**
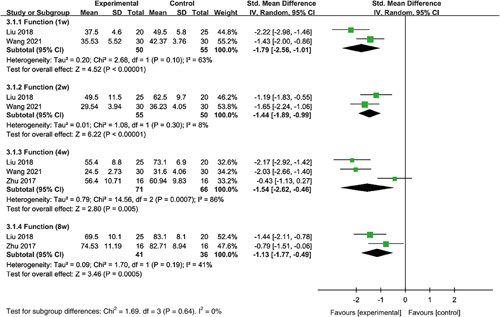
Forest plot of meta-analysis for physical function.

The results of the analyses indicated that duloxetine had a significant improvement in physical function compared with the control group at 1 week (SMD=−1.79, 95% CI: −2.56 to −1.01, *P*<0.00001), 2 weeks (SMD=−1.44, 95% CI: −1.89 to −0.99, *P*<0.00001), 4 weeks (SMD=−1.54, 95% CI: −2.62 to −0.46], *P*=0.005), and 8 weeks (SMD=−1.13, 95% CI: −1.77 to −0.49, *P*=0.0005) (Fig. 5).

#### Analgesics consumption

**Figure 6:**
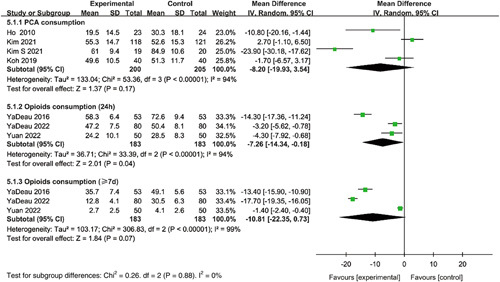
Forest plot of meta-analysis for analgesics consumption.

Meta-analysis of four studies indicated no significant differences between the duloxetine groups and controls for consumption of patient-controlled analgesia with intravenous morphine or fentanyl (MD=−8.20, 95% CI: −19.93 to 3.54, *P*=0.17). Cumulative opioids consumption at 24 h and more than 7 days after TKA were reported in three studies. Results of analyses indicated that the cumulative opioids consumption at 24 h in the duloxetine groups was lower than in the control groups (MD=−7.26, 95% CI: −14.34 to −0.18, *P*=0.04). There was no statistical significance for the cumulative opioid consumption over more than 7 days between the duloxetine groups and controls (MD=−10.81, 95% CI: −22.35 to 0.73, *P*=0.07) (Fig. 6).

### Secondary outcome

#### ROM of knee

**Figure 7:**
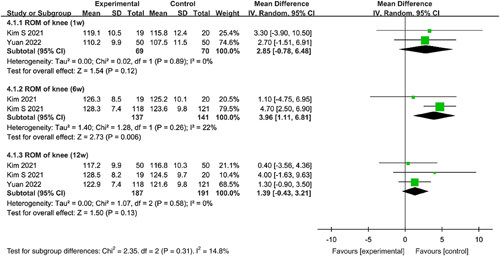
Forest plot of meta-analysis for range of motion (ROM) the of knee.

Meta-analyses were performed to evaluate the effect of duloxetine on ROM of the knee at 1 week, 6, and 12 weeks after TKA. Combined data demonstrated that there were no significant differences in ROM of the knee between the duloxetine groups and controls at 1 week (MD=2.85, 95% CI: −0.78 to 6.48, *P*=0.12) and 12 weeks (MD=1.39, 95% CI: −0.43 to 3.21, *P*=0.13) according to the random-effects model. Duloxetine showed a significant improvement in ROM of the knee compared with the control group at 6 weeks (MD=3.96, 95% CI: 1.11–6.81, *P*=0.006) based on the random-effects model (Fig. 7).

#### Depression

**Figure 8:**
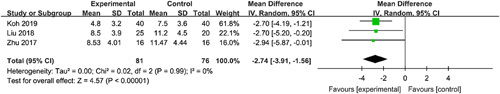
Forest plot of meta-analysis for depression.

Meta-analysis of three studies revealed that patients in the duloxetine group had a lower score on the depression scale than in the control groups with a random-effects model (MD=−2.74, 95% CI: −3.91 to −1.56, *P*<0.00001) (Fig. 8).

#### Mental health

**Figure 9:**
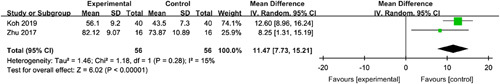
Forest plot of meta-analysis for mental health.

Meta-analysis of two studies reported that patients in the duloxetine group had a better score on the SF-36 for mental health than those in the control groups with a random-effects model (MD=11.47, 95% CI: 7.73–15.21, *P*<0.00001) (Fig. 9).

### Sensitivity analysis

A sensitivity analysis of pain on movement after 1 week was performed by excluding one study at a time to detect the source of heterogeneity. The analysis results indicated that the heterogeneity of pain on movement at 1 week decreased to 35% after excluding the study by Kim and colleagues, which suggested that this study could be the potential source of heterogeneity. Combined data demonstrated that statistical significance still remained in pain on movement at 1 week (SMD=−0.60, 95% CI: −0.93 to −0.28, *P*=0.0003) (Table 2).

**Table 2:** Sensitivity analysis of pain on movement at 1 week.

References	SMD (95% CI)	*P*	*I* ^2^ (%)
Kim *et al.* 23,29	−0.60 (−0.93 to −0.28)	0.0003	35
Koh *et al.* 21	−0.86 (−1.39 to −0.33)	0.001	66
Wang *et al.* 32	−0.66 (−1.11 to −0.21)	0.004	59
Yuan *et al.* 30	−0.88 (−1.35 to −0.41)	0.0003	55

SMD, standard mean difference.

### Publication bias

There was no significant publication bias observed for pain at rest and on movement, physical function, analgesic consumption, ROM of the knee, and depression based on Begg’s and Egger’s tests (Supplementary Table 3, Supplemental Digital Content 4, http://links.lww.com/JS9/A137).

### Quality of evidence

There is moderate evidence in pain at rest at 24 h, 3 days, 6 weeks, and 12 months, pain on movement at 2, 4, 6, 8 weeks and 12 months, physical function at 2 weeks, ROM of the knee, patient-controlled analgesia consumption, depression, and mental health. The evidence of rest outcomes was low based on the GRADE approach to evaluating the quality of the evidence (Supplementary Table 4, Supplemental Digital Content 4, http://links.lww.com/JS9/A137).

### Adverse events

All studies examined adverse events, and no serious adverse events that required specific treatment were reported. Nine studies demonstrated that no significant differences were observed in the incidence of adverse events between the duloxetine groups and controls. One study reported that the percentage of adverse events was high in the duloxetine group. However, one study showed that more patients had constipation and nausea/vomiting in the control group. The details of adverse events for included studies appear in Table 3.

**Table 3:** Adverse events of the included studies

References	Duloxetine group	Control group
Ho *et al.* 20	Headache (1); dizziness (2); nausea/vomiting (3)	Pruritis (1); somnolence (3); dizziness (3); nausea/vomiting (5)
Kim *et al.* 23	Headache (18); drowsiness (22); dizziness (19); nausea/vomiting (14); dry mouth (25); constipation (13)	Headache (23); drowsiness (27); dizziness (21); nausea/vomiting (21); dry mouth (27); constipation (16)
Kim *et al.* 29	Appetite loss (9); nausea/vomiting (3); insomonia (10); dizziness (6); fatigue (7); dry mouth (5); constipation (10)	Appetite loss (8); nausea/vomiting (6); insomonia (7); dizziness (5); fatigue (4); dry mouth (10); constipation (12)
Koh *et al.* 21	Appetite loss (33); nausea/vomiting (13); insomonia (25); dizziness (4); fatigue (34); dry mouth (18); constipation (20)	Appetite loss (37); nausea/vomiting (12); insomonia (27); dizziness (1); fatigue (36); dry mouth (18); constipation (19)
Rienstra *et al.* 24	None reported	Adverse events (12)
Yuan *et al.* 30	Drowsiness (20); nausea/vomiting (5); sweating (2); dizziness (3); fatigue (23); dry mouth (18); constipation (3)	Drowsiness (10); nausea/vomiting (15); sweating (2); dizziness (5); fatigue (26); dry mouth (17); constipation (11)
YaDeau *et al.* 25	Nausea, drowsiness, drowsiness, somnolence, fatigue, nausea, dizziness or hypervigilance (4)	atrial fibrillation, nausea, insomnia, headache or hypervigilance (3)
YaDeau *et al.* 22	Hyponatremia, surgical site blisters, third cranial nerve palsy, kidney stone, hallucinations, diarrhea, diaphoresis, hypertension, decreased urine output, tooth pain or acne (10)	hallucinations, inability to ejaculate, fever with rash at the surgical site or increased pain (4)
Liu^31^	None reported	None reported
Wang *et al.* 32	None reported	None reported
Zhu^33^	Abdominal pain (1); dizziness (3) nausea (2); constipation (1)	Nausea (2); constipation (1); dizziness (1)

Data are presented as number of patients.

### Trial sequential analysis

Trial sequential analysis (TSA) of ROM of the knee, analgesic consumption, depression, and mental health was performed using TSA software version 0.9 (http://www.ctu.dk/tsa) to estimate the required sample size for the statistical power. TSA suggested that the sample size of ROM of the knee and analgesic consumption in our meta-analysis was lower than the required sample size. For the depression and mental health, TSA suggested that the sample size was higher than the required sample size (Supplementary Figs 2–9, Supplemental Digital Content 4, http://links.lww.com/JS9/A137).

## Discussion

Results of this meta-analysis suggested that duloxetine alleviated pain at rest with a time span of 3 days to 6 weeks and pain on movement with a time span of 5 days to 8 weeks. There was no significant association with pain at rest and on movement at 24 h, 12 weeks, 6, and 12 months. We found that duloxetine improved physical function, ROM of knee with a time span of 1–6 weeks, and emotional function (depression and mental health). But it did not help improve the ROM of the knee at 1 week and 12 weeks. Additionally, this meta-analysis showed that duloxetine reduced cumulative opioids consumption at 24 h, but there was no statistical significance for the cumulative opioids consumption over 7 days between the duloxetine groups and controls. Only one study^32^ provided preliminary findings that duloxetine was associated with reduced plasma levels of interleukin-6 and tumor necrosis factor-α. We did not conduct a meta-analysis because of the paucity of additional evidence.

This is the first systematic review and meta-analysis to evaluate the postoperative benefits of duloxetine for patients receiving TKA. A meta-analysis by Branton *et al.* 18 found that duloxetine reduced total postoperative opioid consumption at 24 h and pain after elective orthopedic surgery. However, this study only analyzed outcomes on pain, opioid consumption, and adverse effects. Besides, the study only included six trials with different orthopedic surgeries, two of which were^20^,^25^ for the surgical subtypes of TKA. Additionally, a meta-analysis by Zorrilla-Vaca *et al.* 19 indicated that there was a significant reduction in pain scores at 4, 6, 24, and 48 h and in opioid administration at 24 and 48 h. Nevertheless, this meta-analysis did not assess the effect of duloxetine on postoperative pain in the long term and physical function. Moreover, this study included nine trials with different orthopedic surgeries, only three of which^20^,^21^,^25^ were surgical subtypes of TKA. Although these findings were partly in accordance with the results of our meta-analysis. They did not assess the effect of duloxetine on physical function, ROM of the knee, or emotional function after TKA. We evaluated its effects on pain relief in the short-term and long-term, physical function, ROM of the knee, and emotional function (depression and mental health) after TKA.

TKA is an effective treatment for patients with severe knee osteoarthritis^34^. However, ~20% of patients who undergo TKA are dissatisfied with the overall results of surgery, most of which are for unexplained persistent pain^35^,^36^. It suggested that inadequate postoperative pain control can decline patient satisfaction, quality of life, and physical function, as well as delay rehabilitation^37^. Hence, it is a necessary strategy to integrate pain management into enhanced recovery programs. Because optimized postoperative pain management allows for early mobilization, this is a prerequisite for improving recovery and decreasing the risk of complications. Despite recent advances in multimodal. Pain management that reduce postoperative pain and opioid consumption, the epidemic and over-prescription of opioid continues^38^. In light of this, current multimodal pain management after TKA must be optimized. It has been shown that noxious stimuli resulting from tissue trauma can sensitize peripheral nociceptors, leading to central sensitization (CS)^39^. CS involves various mechanisms, among which a decrease in the descending inhibition pathway plays an important role. The decreased function of the descending inhibitory pathway results in a reduction in serotonin and norepinephrine^40^. Serotonin and norepinephrine are key neurotransmitters in the descending inhibitory pathway and are related to pain control and wound healing^41^. Therefore, some adjuvants such as ketamine, gabapentin, and pregabalin that target CS have been used to manage postoperative pain^42^,^43^. Furthermore, duloxetine is a selective serotonin-norepinephrine dual reuptake inhibitor that increases the levels of both serotonin and norepinephrine and finally enhances the descending inhibitory pathways in the central nervous system^44^. It has been widely approved for depression and anxiety and has been well established for centrally mediated fibromyalgia and chronic musculoskeletal pain^45^,^46^.

As mentioned above, our meta-analysis suggested that duloxetine alleviated pain at rest and during movement over a time span of 3 days to 8 weeks. While there was no significant association at postoperative hours 24 and more than 12 weeks. It might be explained by the fact that there might be an undetectable difference in pain relief between the two groups due to severe pain within 24 h after TKA and less pain at more than 12 weeks postoperatively. The 2-week postoperative period is a critical window during which rehabilitation may improve long-term results^47^. Therefore, reducing pain at rest and during movement during this period could facilitate earlier and longer ambulation and improve postoperative rehabilitation after TKA. We also found that duloxetine improved physical function and emotional function (depression and mental health). A good ROM and sufficient functional improvement are conditions allowing the patients to perform individualized muscle strengthening exercises and to exercise their newly gained mobility^48^. Additionally, depression has a significant impact on hospital length of stay, costs, complications, and readmissions after primary TKA^49^. Therefore, improvement in the outcome measures of pain, physical function, ROM of the knee, depression, and mental health is beneficial to rehabilitation after TKA. Additionally, the results showed duloxetine reduced cumulative opioid consumption at postoperative hour 24, while there was no significant association more than 7 days. The reasons are unclear but may be partly due to variations in the trial designs of the included studies in this meta-analysis. More RCTs are needed to further evaluate these findings. Considering that duloxetine has potential therapeutic value in improving postoperative rehabilitation after TKA, therefore, it is necessary to further determine the optimal time to initiate duloxetine therapy and the optimal duration for postoperative recovery after TKA. Furthermore, no significant differences were observed in the incidence of adverse events between the duloxetine groups and controls. Nevertheless, more research is needed to support the safety of duloxetine in patients undergoing TKA. Outcome measures, including recovery time and the patient’s fear of joint movement after surgery, were not included in the included studies. These were vital components that need to be included in future studies.

This meta-analysis has several limitations. First, we attempted to perform prespecified subgroup analyses based on the different control groups. However, after performing the data analysis we found that the number of studies did not support the subgroup analysis. Limited studies may lead to bias given the small study effects. Therefore, some conclusions should be considered preliminary. Second, some factors related to heterogeneity remained uncertain despite the fact that we explained heterogeneity to some extent through sensitivity analyses. Considerable heterogeneity remained unclear. This might be associated with the differences in duloxetine intervention characteristics (e.g. dose, start time of administration, and duration) and the measuring method of outcomes. Additionally, the dosage of duloxetine is still not standard. We recommend establishing a guideline for duloxetine prescribing for postoperative recovery of TKA to minimize heterogeneity between studies. Third, limiting the inclusion criteria to RCTs may bias the results. Large-scale and rigorous RCTs are still required in the future.

## Conclusion

In conclusion, duloxetine might reduce pain mainly over a time span of 3 days to 8 weeks and lower cumulative opioid consumption within 24 h. In addition, it improved physical function, ROM of the knee with a time span of 1–6 weeks, and emotional function (depression and mental health).

## Ethical approval

This study did not require ethical approval.

## Sources of funding

None.

## Author contribution

J.-M.Y. and Y.W. contributed equally to the work as first authors. J.-M.Y. and Z.-H.C. had full access to all of the data in this study. J.-M.Y., Y.W., and Z.-H.C.: study design. Z.-H.C., L.O. and Z.S.: scientific advisor. J.-M.Y., Y.W., J.-Y.L.: literature search. J.-Y.L., C.-C.L., and Z.-T.W.: data extraction. J.-M.Y., C.-C.L., Z.-T.W., and Z.-H.C.: statistical analyses. J.-M.Y., Y.W., J.-Y.L.: writing. Z.S. and Z.-H.C.: language editing. All authors revised the article for important content and approved the final version for the article.

## Conflicts of interest disclosure

The authors declare no relevant conflict of interest.

## Research registration unique identifying number (UIN)

1. Name of the registry: PROSPERO.
2. Unique identifying number or registration ID: CRD42022352003.
3. Hyperlink to your specific registration (must be publicly accessible and will be checked): https://www.crd.york.ac.uk/prospero/display_record.php?ID=CRD42022352003

## Guarantor

Jia-Man Yang and Ze-Hua Chen.

## Data statement

The authors confirm that the data supporting the findings of this study are available within the article [and/or] its supplementary materials.

## Provenance and peer review

Not commissioned, externally peer-reviewed.

## Supplementary Material
