# Supplementary figures and images for: Duloxetine for rehabilitation after total knee arthroplasty: a systematic review and meta-analysis
Source: Int J Surg. 2023 Mar 15;109(4):913–24. doi: 10.1097/JS9.0000000000000230 (PMC10389646; doi:10.1097/JS9.0000000000000230)

PRISMA flowchart of study selection
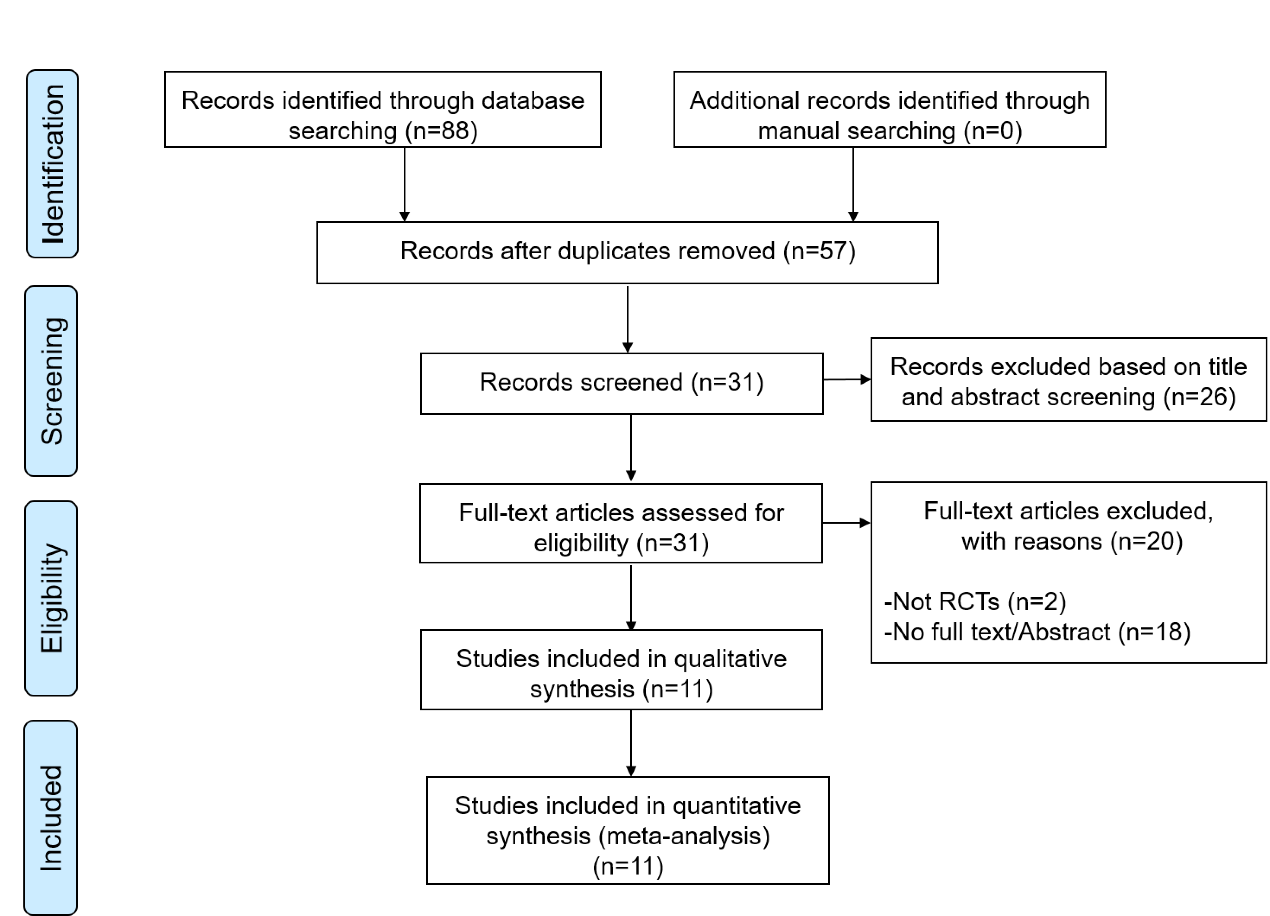

Supplement: Supplementary file 1 [file js9-109-0913-s001.docx]

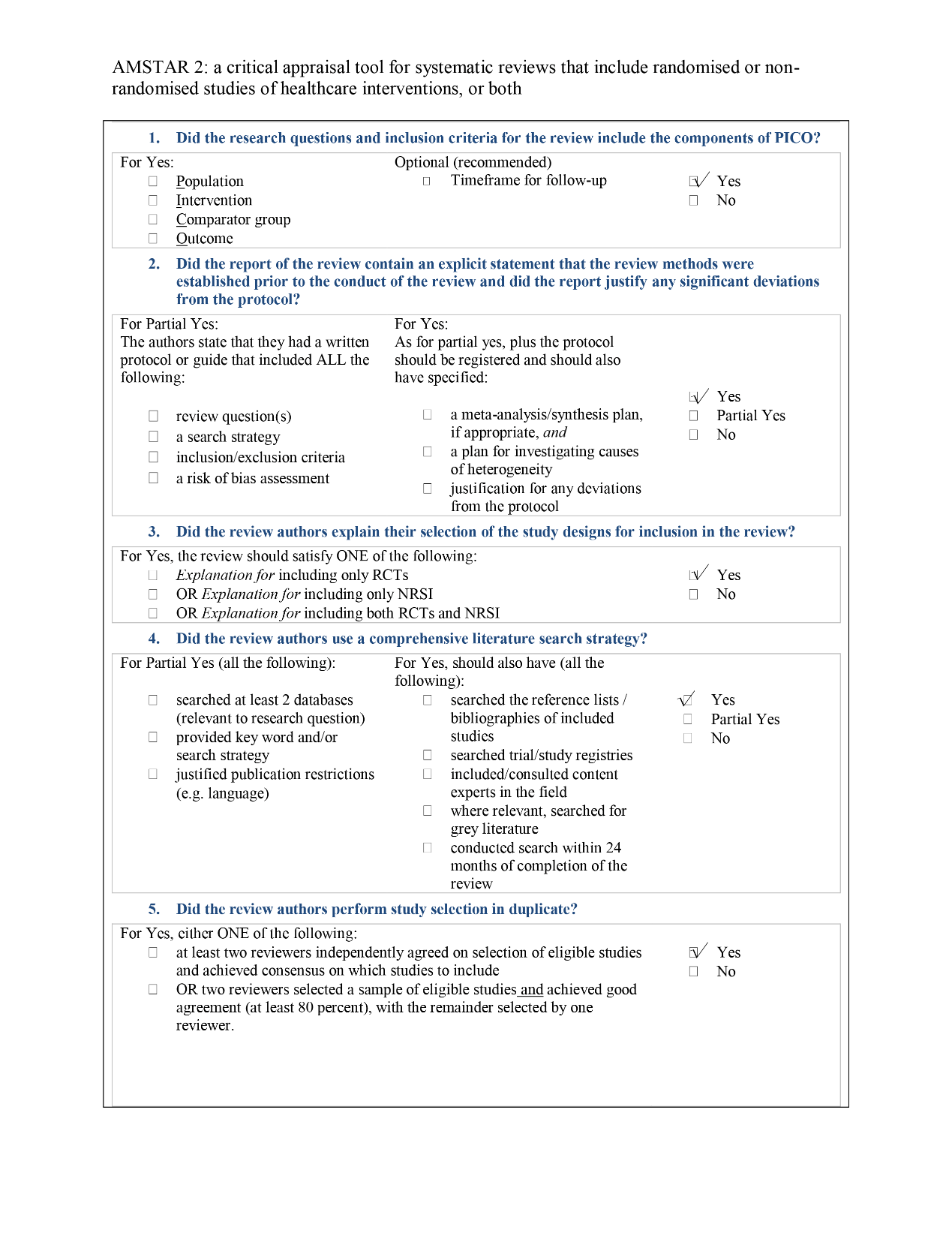


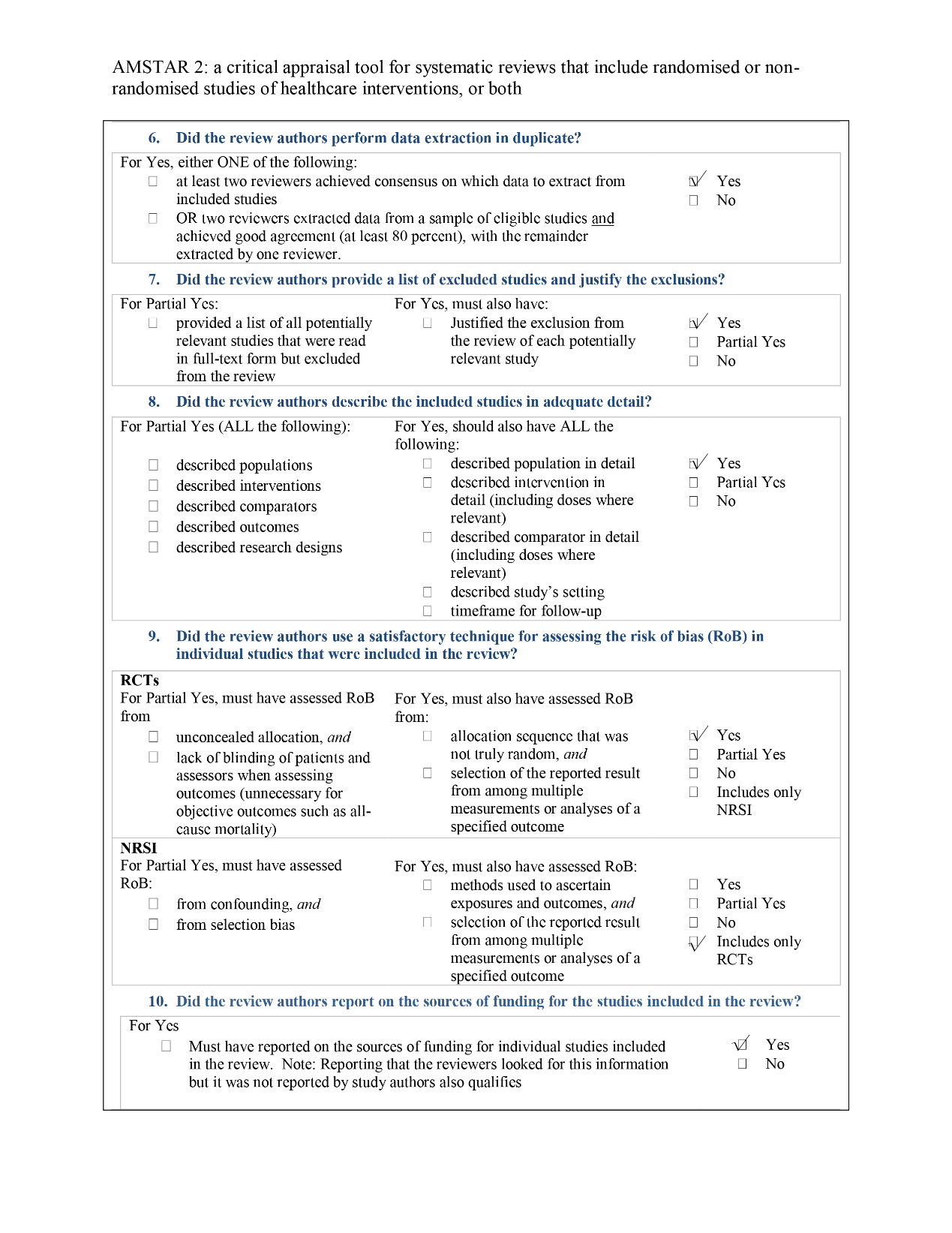


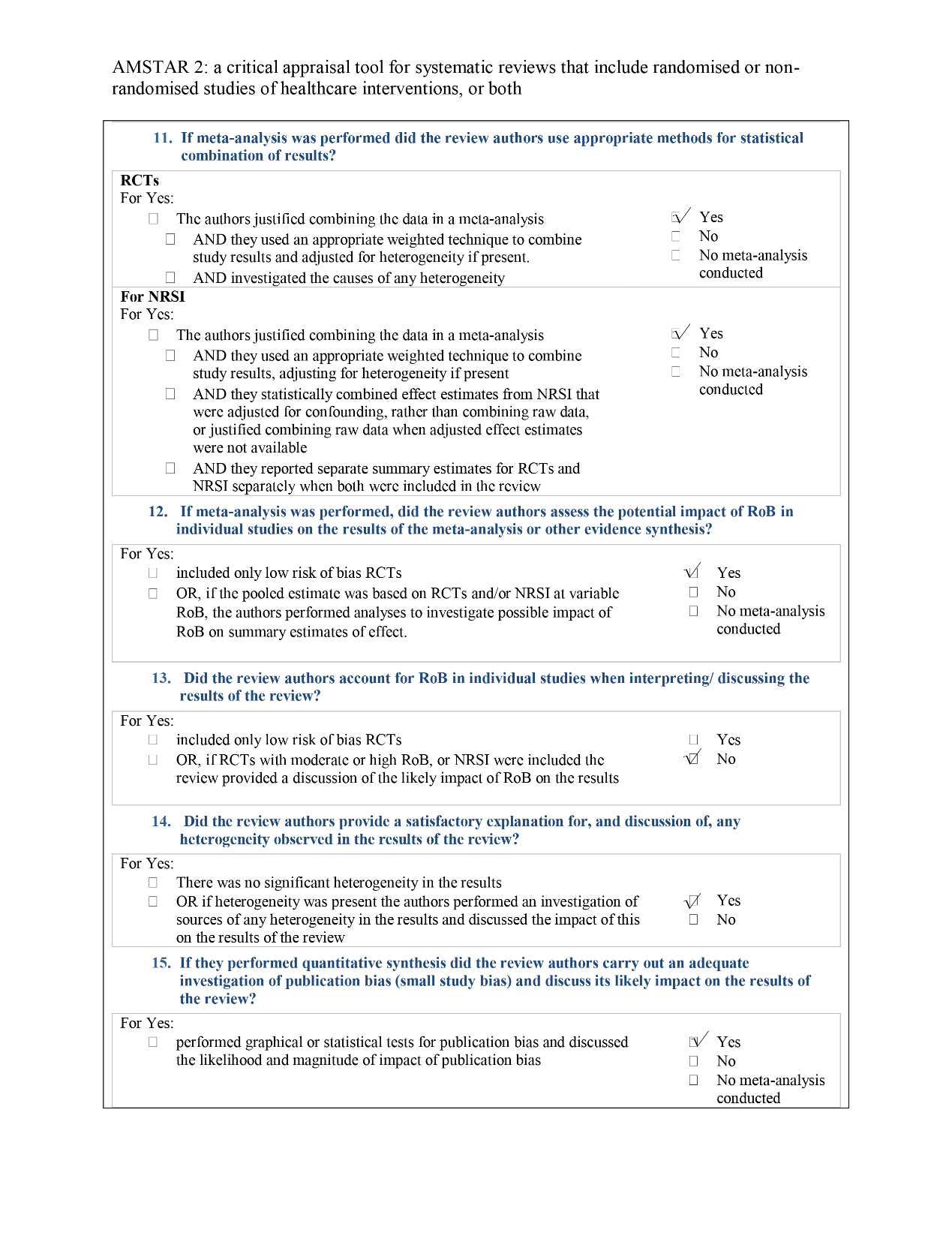


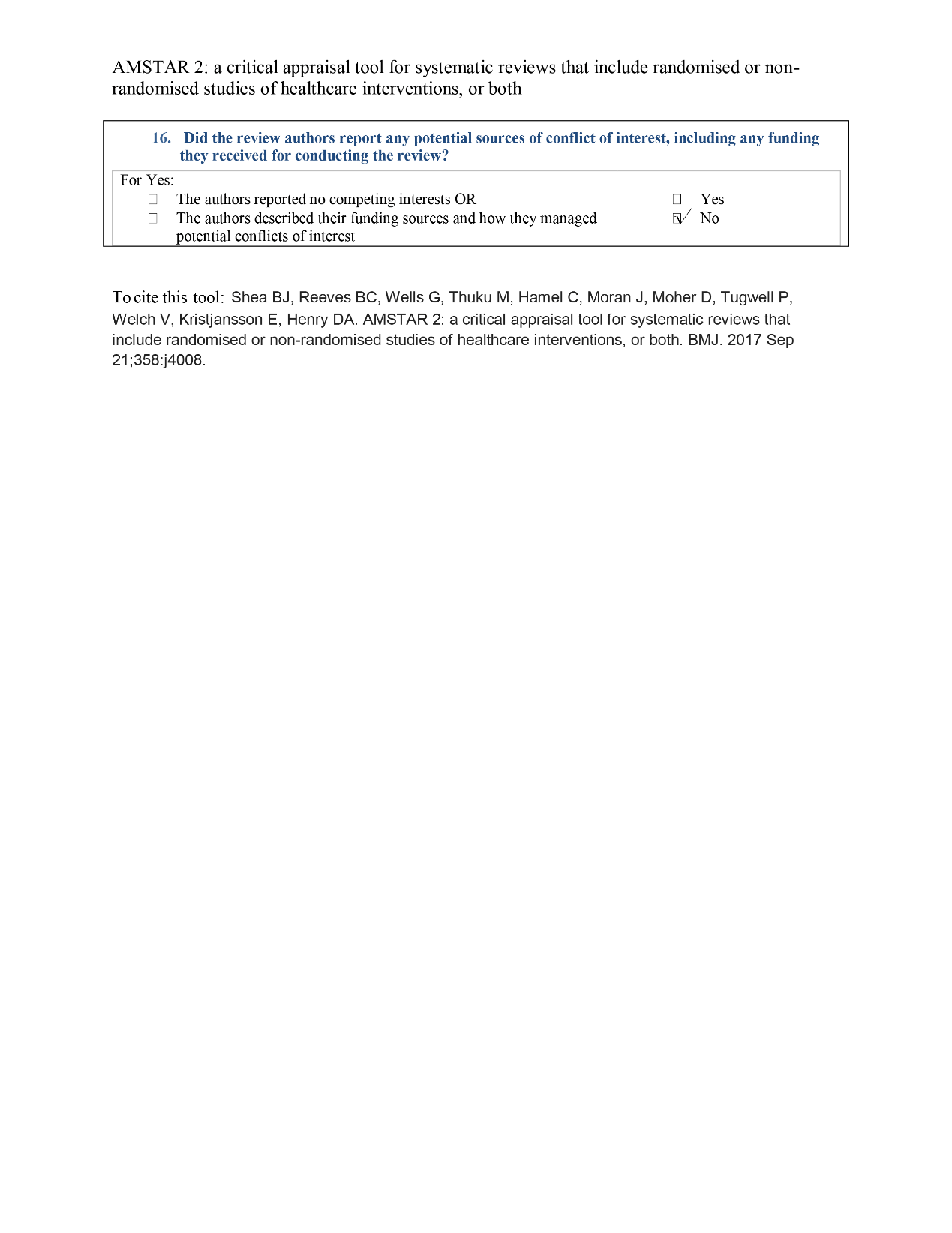

Supplement: Supplementary file 3 [file js9-109-0913-s003.docx]
