# Supplementary material for: Duloxetine for rehabilitation after total knee arthroplasty: a systematic review and meta-analysis
Source: Int J Surg. 2023 Mar 15;109(4):913–24. doi: 10.1097/JS9.0000000000000230 (PMC10389646; doi:10.1097/JS9.0000000000000230)
Supplement: Supplementary file 4 [file js9-109-0913-s004.docx]

**Supplementary Material**

**Table S1** Search strategy used

**Table S2** Excluded Trials and Reasons for Exclusion

**Table S3** Assessment of publication bias

**Table S4** Evidence quality assessment according to GRADE

**Table S5** AMSTAR checklist

**Table S6** PRISMA 2020 checklist

**Figure F1** PRISMA flowchart of study selection

**Figure F2** Trial sequential analysis for ROM of knee at 1 week

**Figure F3** Trial sequential analysis for ROM of knee at 6 weeks

**Figure F4** Trial sequential analysis for ROM of knee at 12 weeks

**Figure F5** Trial sequential analysis for patient-controlled analgesia consumption

**Figure F6** Trial sequential analysis for opioids consumption at 24 h

**Figure F7** Trial sequential analysis for opioids consumption more than 7 days

**Figure F8** Trial sequential analysis for depression

**Figure F9** Trial sequential analysis for mental health

This supplementary material has been provided by the authors to give readers additional

information about their work.

**Table S1 - Search strategy used**

| Pubmed | #1 ((((((((((((((("Duloxetine Hydrochloride"[Mesh]) OR (Hydrochloride, Duloxetine[Title/Abstract])) OR (Duloxetine HCl[Title/Abstract])) OR (HCl, Duloxetine[Title/Abstract])) OR (LY 248686[Title/Abstract])) OR (LY-248686[Title/Abstract])) OR (LY248686[Title/Abstract])) OR (Duloxetine Ethanedioate (1:1), (+-)-isomer - T353987[Title/Abstract])) OR (LY 227942[Title/Abstract])) OR (LY-227942[Title/Abstract])) OR (LY227942[Title/Abstract])) OR (Duloxetine[Title/Abstract])) OR (N-methyl-3-(1-naphthalenyloxy)-3-(2-thiophene)propanamide[Title/Abstract])) OR (N-methyl-3-(1-naphthalenyloxy)-2-thiophenepropanamine[Title/Abstract])) OR (Duloxetine, (+) isomer[Title/Abstract])) OR (Cymbalta[Title/Abstract]) 3078  #2 ("Arthroplasty, Replacement, Knee"[Mesh]) OR ((Arthroplasties, Replacement, Knee[Title/Abstract])) OR (Arthroplasty, Knee Replacement[Title/Abstract])) OR (Knee Replacement Arthroplasties[Title/Abstract])) OR (Knee Replacement Arthroplasty[Title/Abstract])) OR (Replacement Arthroplasties, Knee[Title/Abstract])) OR (Knee Arthroplasty, Total[Title/Abstract])) OR (Arthroplasty, Total Knee[Title/Abstract])) OR (Total Knee Arthroplasty[Title/Abstract])) OR (Replacement, Total Knee[Title/Abstract])) OR (Total Knee Replacement[Title/Abstract])) OR (Knee Replacement, Total[Title/Abstract])) OR (Knee Arthroplasty[Title/Abstract])) OR (Arthroplasty, Knee[Title/Abstract])) OR (Arthroplasties, Knee Replacement[Title/Abstract])) OR (Replacement Arthroplasty, Knee[Title/Abstract])) OR (Arthroplasty, Replacement, Partial Knee[Title/Abstract])) OR (Unicompartmental Knee Arthroplasty[Title/Abstract])) OR (Arthroplasty, Unicompartmental Knee[Title/Abstract])) OR (Knee Arthroplasty, Unicompartmental[Title/Abstract])) OR (Unicondylar Knee Arthroplasty[Title/Abstract])) OR (Arthroplasty, Unicondylar Knee[Title/Abstract])) OR (Knee Arthroplasty, Unicondylar[Title/Abstract])) OR (Partial Knee Arthroplasty[Title/Abstract])) OR (Arthroplasty, Partial Knee[Title/Abstract])) OR (Knee Arthroplasty, Partial[Title/Abstract])) OR (Unicondylar Knee Replacement[Title/Abstract])) OR (Knee Replacement, Unicondylar[Title/Abstract])) OR (Partial Knee Replacement[Title/Abstract])) OR (Knee Replacement, Partial[Title/Abstract])) OR (Unicompartmental Knee Replacement[Title/Abstract])) OR (Knee Replacement, Unicompartmental[Title/Abstract]))  40,672  #3 (((((((("Randomized Controlled Trial" [Publication Type]) OR (Randomized Controlled Trial[Title/Abstract])) OR (controlled clinical trial[Publication Type])) OR (randomly [Title/Abstract])) OR (placebo[Title/Abstract])) OR (randomized[Title/Abstract])) OR (trial[Title/Abstract])) OR (groups[Title/Abstract])) NOT (animals [mh] NOT humans [mh]))  3,013,846  #4 #1 AND #2 AND #3 11 |
| --- | --- |
| Embase | #1 'duloxetine'/exp 12,818  #2 '1 naphthyloxy':ab,ti AND 3:ab,ti AND '2 thienyl':ab,ti AND 'n methylpropylamine':ab,ti OR ('naphth 1 yloxy':ab,ti AND 3:ab,ti AND 'thien 2 yl':ab,ti AND 'n methylpropylamine':ab,ti) OR ariclaim:ab,ti OR cymbalta:ab,ti OR 'dlx iso3':ab,ti OR dlxiso3:ab,ti OR drizalma:ab,ti OR duloxetine:ab,ti OR dulane:ab,ti OR 'duloxetine boehringer ingelheim':ab,ti OR 'duloxetine hydrochloride':ab,ti OR duzela:ab,ti OR 'ly 248686':ab,ti OR ly248686:ab,ti OR ('n methyl 3':ab,ti AND '1 naphthalenyloxy':ab,ti AND '2 thiophenepropanamine':ab,ti) OR ('n methyl 3':ab,ti AND '1 naphthalenyloxy':ab,ti AND 3:ab,ti AND '2 thiophenyl':ab,ti AND '1 propanamine':ab,ti) OR ('n methyl 3':ab,ti AND '1 naphthyloxy':ab,ti AND 3:ab,ti AND '2 thienyl':ab,ti AND propylamine:ab,ti) OR ('n methyl 3':ab,ti AND 'naphth 1 yloxy':ab,ti AND 3:ab,ti AND 'thien 2 yl':ab,ti AND propylamine:ab,ti) OR ('n methyl 3':ab,ti AND 'naphthalen 1 yloxy':ab,ti AND '2 thiophenepropanamine':ab,ti) OR ('n methyl 3':ab,ti AND 'naphthalen 1 yloxy':ab,ti AND 3:ab,ti AND 'thiophen 2 yl':ab,ti AND 'propan 1 amine':ab,ti) OR nodetrip:ab,ti OR xeristar:ab,ti OR yentreve:ab,ti 4,845  #3 #1 OR #2 13,090  #4 'total knee arthroplasty'/exp 33,131  #5 'knee arthroplasty, total':ab,ti OR 'knee replacement, total':ab,ti OR 'total knee joint replacement':ab,ti OR 'total knee replacement':ab,ti OR 'total knee replacement arthroplasty':ab,ti  8,241  #6 #4 OR #5 35,131  #7 ('randomized controlled trial'/exp OR 'controlled trial, randomized':ab,ti OR 'randomised controlled study':ab,ti OR 'randomised controlled trial':ab,ti OR 'randomized controlled study':ab,ti OR 'trial, randomized controlled':ab,ti) 732,394  #8 #3 AND #6 AND #7 9 |
| Web of Science | #1 TS=(Duloxetine Hydrochloride OR Hydrochloride, Duloxetine OR Duloxetine OR Duloxetine HCl OR HCl, Duloxetine OR LY 248686 OR LY248686 OR LY248686 OR Duloxetine Ethanedioate (1:1), (+-)-isomer - T353987 OR LY 227942 OR LY-227942 OR LY227942 OR N-methyl-3-(1-naphthalenyloxy)-3-(2-thiophene)propanamide OR N-methyl-3-(1-naphthalenyloxy)-2-thiophenepropanamine OR Duloxetine, (+)-isomer OR Cymbalta) 5199  #2 TS=(Arthroplasty, Replacement, Knee OR Arthroplasties, Replacement, Knee OR Arthroplasty, Knee Replacement OR Knee Replacement Arthroplasties OR Knee Replacement Arthroplasty OR Replacement Arthroplasties, Knee OR Knee Arthroplasty, Total OR Arthroplasty, Total Knee OR Total Knee Arthroplasty OR Replacement, Total Knee OR Total Knee Replacement OR Knee Replacement, Total OR Knee Arthroplasty OR Arthroplasty, Knee OR Arthroplasties, Knee Replacement OR Replacement Arthroplasty, Knee OR Arthroplasty, Replacement, Partial Knee OR Unicompartmental Knee Arthroplasty OR Arthroplasty, Unicompartmental Knee OR Knee Arthroplasty, Unicompartmental OR Unicondylar Knee Arthroplasty OR Arthroplasty, Unicondylar Knee OR Knee Arthroplasty, Unicondylar OR Partial Knee Arthroplasty OR Arthroplasty, Partial Knee OR Knee Arthroplasty, Partial OR Unicondylar Knee Replacement OR Knee Replacement, Unicondylar OR Partial Knee Replacement OR Knee Replacement, Partial OR Unicompartmental Knee Replacement OR Knee Replacement, Unicompartmental) 62,650  #3 TS=(Randomized Controlled Trial OR controlled clinical trial OR randomized OR placebo OR randomly OR trial) [2,946,981](http://www--webofscience--com--https.webofknowledge.gzzyy.yuntsg.cn:2222/wos/alldb/summary/40ce81ad-88b2-46d7-9945-5b14820ff97b-4284f9f7/relevance/1)  #4 #1 AND #2 AND #3 27 |
| Cochrane Library | #1 MeSH descriptor: [T Duloxetine Hydrochloride] explode all trees 603  #2 (Duloxetine Ethanedioate):ti,ab,kw OR (HCl, Duloxetine):ti,ab,kw OR (Duloxetine HCl):ti,ab,kw OR (Hydrochloride, Duloxetine):ti,ab,kw OR (LY-227942):ti,ab,kw OR (LY 227942):ti,ab,kw OR (LY227942):ti,ab,kw OR (Duloxetine):ti,ab,kw (Word variations have been searched) OR (Cymbalta):ti,ab,kw OR (LY-248686):ti,ab,kw OR (LY 248686):ti,ab,kw OR (LY248686):ti,ab,kw 2,303  #3 #1 OR #2 1,566  #4 MeSH descriptor: [Arthroplasty, Replacement, Knee] explode all trees 2,898  #5 (Knee Replacement, Partial):ti,ab,kw OR (Partial Knee Replacement):ti,ab,kw OR (Partial Knee Arthroplasty):ti,ab,kw OR (Unicondylar Knee Replacement):ti,ab,kw OR (Knee Arthroplasty, Partial):ti,ab,kw OR (Arthroplasty, Partial Knee):ti,ab,kw OR (Unicompartmental Knee Replacement):ti,ab,kw OR (Knee Arthroplasty, Unicondylar):ti,ab,kw OR (Unicondylar Knee Replacement):ti,ab,kw OR (Unicompartmental Knee Arthroplasty):ti,ab,kw OR (Arthroplasty, Unicompartmental Knee):ti,ab,kw OR (Arthroplasty, Replacement, Partial Knee):ti,ab,kw OR (Knee Replacement, Unicondylar):ti,ab,kw OR (Knee Replacement, Unicompartmental):ti,ab,kw OR (Knee Arthroplasty, Unicompartmental):ti,ab,kw OR (Total Knee Arthroplasty):ti,ab,kw OR (Knee Arthroplasty):ti,ab,kw OR (Replacement Arthroplasty, Knee):ti,ab,kw OR (Total Knee Replacement):ti,ab,kw OR (Replacement, Total KneeReplacement, Total Knee):ti,ab,kw OR (Knee Replacement Arthroplasty):ti,ab,kw OR (Replacement Arthroplasties, Knee):ti,ab,kw OR (Arthroplasty, Knee):ti,ab,kw OR (Arthroplasties, Replacement, Knee):ti,ab,kw OR (Knee Replacement, Total):ti,ab,kw OR (Arthroplasty, Total Knee):ti,ab,kw OR (Knee Arthroplasty, Total):ti,ab,kw OR (Arthroplasty, Knee Replacement):ti,ab,kw OR (Arthroplasties, Knee Replacement):ti,ab,kw OR (Knee Replacement Arthroplasties):ti,ab,kw 17,623  #6 #4 OR #5 9,110  #7 #3 AND #6 35 |
| CNKI | (主题：度洛西汀 (精确)) AND ((主题：全膝关节置换术 (精确)) OR (主题：关节置换（精确）) OR (主题：关节置换术 (精确))) AND ((摘要：随机对照 (精确)) OR (摘要：随机 (精确))OR(摘要：RCT (精确))) |
| VIP | (M=(度洛西汀) AND (M=全膝关节置换术 OR 关节置换术 OR 关节置换 ) AND (R=随机对照 OR 随机 OR RCT) |
| Wanfang | 主题:(度洛西汀) and 主题:(全膝关节置换术 or 关节置换术 or 关节置换 ) and 主题:(随机对照 or 随机 or RCT) |
| SinoMed | 1 "度洛西汀"[常用字段:智能] 3963  2 "全膝关节置换术"[常用字段:智能] OR "关节置换术"[常用字段:智能] OR "关节置换"[常用字段:智能] 44658  3 "随机对照"[摘要:智能] OR "随机"[摘要:智能] OR "RCT"[摘要:智能] 1800213  4 1 AND 2 AND 3 1 |

**Table S2 - Excluded Trials and Reasons for Exclusion**

| **Excluded trials** | **Reason for exclusion** |
| --- | --- |
| Blikman 2015^1^ | This is an ongoing study, and no relevent data is available now |
| ChiCtr 2019 ^2^ | This is an ongoing study, and no relevent data is available now |
| ChiCtr 2019^3^ | This is an ongoing study, and no relevent data is available now |
| ChiCtr 2020^4^ | This is an ongoing study, and no relevent data is available now |
| ChiCtr 2020^5^ | This is an ongoing study, and no relevent data is available now |
| Euctr 2014^6^ | This is an ongoing study, and no relevent data is available now |
| Irct 2020^7^ | This is an ongoing study, and no relevent data is available now |
| Jacques 2016^8^ | This is an ongoing study, and no relevent data is available now |
| Nct 2013^9^ | This is an ongoing study, and no relevent data is available now |
| Nct 2014^10^ | This is an ongoing study, and no relevent data is available now |
| Nct 2015^11^ | This is an ongoing study, and no relevent data is available now |
| Nct 2017^12^ | This is an ongoing study, and no relevent data is available now |
| Nct 2019^13^ | This is an ongoing study, and no relevent data is available now |
| Nct 2019^14^ | This is an ongoing study, and no relevent data is available now |
| Nct 2021^15^ | This is an ongoing study, and no relevent data is available now |
| Nct 2021^16^ | This is an ongoing study, and no relevent data is available now |
| Ntr 2014^17^ | This is an ongoing study, and no relevent data is available now |
| Tctr 2020^18^ | This is an ongoing study, and no relevent data is available now |
| Jacobs 2016^19^ | This is not a randomized controlled trial |
| Kazarian 2021^20^ | This is not a randomized controlled trial |

**REFERENCES**

1. Blikman T, Rienstra W, van Raaij TM, Bulstra SK, Stevens M, van den Akker-Scheek I. THE EFFECT OF PREOPERATIVE PAIN TREATMENT BY MEANS OF DULOXETINE ON POSTOPERATIVE OUTCOME AFTER TOTAL HIP OR KNEE ARTHROPLASTY: DESIGN OF A PRAGMATIC RANDOMIZED CONTROLLED TRIAL. *OSTEOARTHRITIS AND CARTILAGE.* 2015;23:A361-A361.

2. ChiCtr. Role of SERTRALINE (SSRI) Versus DULOXETINE (SSNRI) among depressed total knee arthroplasty candidates: a prospective, randomized, patient selective, double blind study. [*https://trialsearchwhoint/Trial2aspx?TrialID=ChiCTR1900027004*](https://trialsearchwhoint/Trial2aspx?TrialID=ChiCTR1900027004)*.* 2019.

3. ChiCtr. Compare the effect of SERTRALINE (SSRI) Versus DULOXETINE (SSNRI) among depressed total hip arthroplasty candidates: a prospective, randomized, patient selective, double blind study. [*https://trialsearchwhoint/Trial2aspx?TrialID=ChiCTR1900027005*](https://trialsearchwhoint/Trial2aspx?TrialID=ChiCTR1900027005)*.* 2019.

4. ChiCtr. The Effects of Duloxetine on Pain and Hip Function after Total Knee Arthroplasty: a Randomized Controlled Trial. [*https://trialsearchwhoint/Trial2aspx?TrialID=ChiCTR2000033910*](https://trialsearchwhoint/Trial2aspx?TrialID=ChiCTR2000033910)*.* 2020.

5. ChiCtr. The Effects of Duloxetine on Pain and Emotional Function Following Total Knee Arthroplasty in Patients with Anxiety and Depression: a Randomized Controlled Trial. [*https://trialsearchwhoint/Trial2aspx?TrialID=ChiCTR2000033903*](https://trialsearchwhoint/Trial2aspx?TrialID=ChiCTR2000033903)*.* 2020.

6. Euctr NL. Effect of pre-operative pain treatment on postoperative outcome after total hip or knee replacement surgery. [*https://trialsearchwhoint/Trial2aspx?TrialID=EUCTR2013-004313-41-NL*](https://trialsearchwhoint/Trial2aspx?TrialID=EUCTR2013-004313-41-NL)*.* 2014.

7. Irct20190719044276N. The comparison of effect of pregabalin and duloxetine on post-op pain. [*https://trialsearchwhoint/Trial2aspx?TrialID=IRCT20190719044276N1*](https://trialsearchwhoint/Trial2aspx?TrialID=IRCT20190719044276N1)*.* 2020.

8. Jacques YD. Does duloxetine reduce sub-acute pain after knee arthroplasty? A randomized controlled trial. *Regional Anesthesia and Pain Medicine.* 2016;41(5).

9. Nct. Does Duloxetine Reduce Sub-Acute Pain After Knee Arthroplasty? [*https://clinicaltrialsgov/show/NCT02005601*](https://clinicaltrialsgov/show/NCT02005601)*.* 2013.

10. Nct. Does Duloxetine Reduce Chronic Pain After Total Knee Arthroplasty? [*https://clinicaltrialsgov/show/NCT02307305*](https://clinicaltrialsgov/show/NCT02307305)*.* 2014.

11. Nct. Effects of Duloxetine on Pain Relief After Total Knee Arthroplasty in Central Sensitization Patient. [*https://clinicaltrialsgov/show/NCT02600247*](https://clinicaltrialsgov/show/NCT02600247)*.* 2015.

12. Nct. Effect of Duloxetine on Opioid Use After Total Knee Arthroplasty. [*https://clinicaltrialsgov/show/NCT03271151*](https://clinicaltrialsgov/show/NCT03271151)*.* 2017.

13. Nct. Pre-emptive Effect of Duloxetine in the Second Knee in Staged Total Knee Arthroplasty. [*https://clinicaltrialsgov/show/NCT03792828*](https://clinicaltrialsgov/show/NCT03792828)*.* 2019.

14. Nct. Effects of Duloxetine on Postoperative Wound Complication of Total Knee Arthroplasty (TKA) in Central Sensitization Patients. [*https://clinicaltrialsgov/show/NCT03880916*](https://clinicaltrialsgov/show/NCT03880916)*.* 2019.

15. Nct. Comparison of Opioid and Duloxetine for Postoperative Pain Control After Total Knee Arthroplasty: RCT. [*https://clinicaltrialsgov/show/NCT04719585*](https://clinicaltrialsgov/show/NCT04719585)*.* 2021.

16. Nct. Duloxetine RCT on Postop TKA Outcomes. [*https://clinicaltrialsgov/show/NCT05086393*](https://clinicaltrialsgov/show/NCT05086393)*.* 2021.

17. Ntr. Effect of pre-operative pain treatment by means of duloxetine on postoperative outcome after total hip or knee arthroplasty- The DOA study. [*https://trialsearchwhoint/Trial2aspx?TrialID=NTR4744*](https://trialsearchwhoint/Trial2aspx?TrialID=NTR4744)*.* 2014.

18. Tctr. Does duloxetine reduces pain in total knee arthroplasty? Randomized controlled trial. [*https://trialsearchwhoint/Trial2aspx?TrialID=TCTR20200925002*](https://trialsearchwhoint/Trial2aspx?TrialID=TCTR20200925002)*.* 2020.

19. Jacobs MB, Cohen SP. Duloxetine for Subacute Pain Management after Total Knee Arthroplasty Should We Write It Off or Reevaluate? *Anesthesiology.* 2016;125(3):454-456.

20. Kazarian GS, Anthony CA, Lawrie CM, Barrack RL. The Impact of Psychological Factors and Their Treatment on the Results of Total Knee Arthroplasty. *JOURNAL OF BONE AND JOINT SURGERY-AMERICAN VOLUME.* 2021;103(18):1744-1756.

**Table S3 - Assessment of publication bias**

| **Outcomes** | **Begg' s test** | **Egger's test** |
| --- | --- | --- |
| Pain at rest at 24 h | 0.308 | 0.060 |
| Pain at rest at 3 d | 0.296 | 0.071 |
| Pain at rest at 1 w | 1.000 | 0.260 |
| Pain at rest at 2 w | 1.000 | 0.280 |
| Pain at rest at 6 w | 0.734 | 0.145 |
| Pain at rest at 12 w | 1.000 | 0.350 |
| Pain on movement at 24 h | 0.308 | 0.072 |
| Pain on movement at 1w | 0.089 | 0.082 |
| Pain on movement at 2 w | 1.000 | 0.060 |
| Pain on movement at 4 w | 1.000 | 0.966 |
| Pain on movement at 6 w | 0.806 | 0.248 |
| Pain on movement at 12 w | 1.000 | 0.355 |
| Physical function at 4 w | 1.000 | 0.917 |
| ROM of knee at 12 w | 1.000 | 0.621 |
| PCA consumption | 0.089 | 0.060 |
| Opioids consumption at 24h | 0.296 | 0.407 |
| Opioids consumption ≥7d | 0.296 | 0.220 |
| Depression | 1.000 | 0.406 |

**Table S4 - Evidence quality assessment according to GRADE**

| **Duloxetine for TKA** | | | | | | |
| --- | --- | --- | --- | --- | --- | --- |
| **Patient or population:** patients with TKA **Settings:**  **Intervention:** Duloxetine | | | | | | |
| **Outcomes** | **Illustrative comparative risks* (95% CI)** | | **Relative effect (95% CI)** | **No of Participants (studies)** | **Quality of the evidence (GRADE)** | **Comments** |
|  | Assumed risk | Corresponding risk |  |  |  |  |
|  | **Control** | **Duloxetine** |  |  |  |  |
| **Pain at rest - Pain (24h)** |  | The mean pain at rest - pain (24h) in the intervention groups was **0.43 standard deviations lower** (1.06 lower to 0.2 higher) |  | 379 (4 studies) | ⊕⊕⊕⊝ **moderate**^1^ | SMD -0.43 (-1.06 to 0.2) |
| **Pain at rest - Pain (3d)** |  | The mean pain at rest - pain (3d) in the intervention groups was **0.35 standard deviations lower** (0.65 to 0.05 lower) |  | 225 (3 studies) | ⊕⊕⊕⊝ **moderate**^1^ | SMD -0.35 (-0.65 to -0.05) |
| **Pain at rest - Pain (1w)** |  | The mean pain at rest - pain (1w) in the intervention groups was **0.56 standard deviations lower** (1.02 to 0.1 lower) |  | 219 (3 studies) | ⊕⊕⊝⊝ **low**^1,2^ | SMD -0.56 (-1.02 to -0.1) |
| **Pain at rest - Pain (2w)** |  | The mean pain at rest - pain (2w) in the intervention groups was **0.73 standard deviations lower** (1.31 to 0.14 lower) |  | 279 (3 studies) | ⊕⊕⊝⊝ **low**^1,2^ | SMD -0.73 (-1.31 to -0.14) |
| **Pain at rest - Pain (6w)** |  | The mean pain at rest - pain (6w) in the intervention groups was **0.78 standard deviations lower** (1.36 to 0.21 lower) |  | 464 (4 studies) | ⊕⊕⊕⊝ **moderate**^2^ | SMD -0.78 (-1.36 to -0.21) |
| **Pain at rest - Pain (12w)** |  | The mean pain at rest - pain (12w) in the intervention groups was **0.36 standard deviations lower** (0.85 lower to 0.13 higher) |  | 358 (3 studies) | ⊕⊕⊝⊝ **low**^1,2^ | SMD -0.36 (-0.85 to 0.13) |
| **Pain at rest - Pain (6m)** |  | The mean pain at rest - pain (6m) in the intervention groups was **0.16 standard deviations higher** (0.57 lower to 0.89 higher) |  | 350 (2 studies) | ⊕⊕⊝⊝ **low**^1,2^ | SMD 0.16 (-0.57 to 0.89) |
| **Pain at rest - Pain (12m)** |  | The mean pain at rest - pain (12m) in the intervention groups was **0.19 standard deviations lower** (0.4 lower to 0.02 higher) |  | 350 (2 studies) | ⊕⊕⊕⊝ **moderate**^1^ | SMD -0.19 (-0.4 to 0.02) |
| **Pain on movement - Pain (24h)** |  | The mean pain on movement - pain (24h) in the intervention groups was **0.46 standard deviations lower** (0.96 lower to 0.03 higher) |  | 379 (4 studies) | ⊕⊕⊝⊝ **low**^1,2^ | SMD -0.46 (-0.96 to 0.03) |
| **Pain on movement - Pain (5d)** |  | The mean pain on movement - pain (5d) in the intervention groups was **0.65 standard deviations lower** (1.21 to 0.09 lower) |  | 119 (2 studies) | ⊕⊕⊝⊝ **low**^1,2^ | SMD -0.65 (-1.21 to -0.09) |
| **Pain on movement - Pain (1w)** |  | The mean pain on movement - pain (1w) in the intervention groups was **0.74 standard deviations lower** (1.12 to 0.36 lower) |  | 279 (4 studies) | ⊕⊕⊝⊝ **low**^1,2^ | SMD -0.74 (-1.12 to -0.36) |
| **Pain on movement - Pain (2w)** |  | The mean pain on movement - pain (2w) in the intervention groups was **0.83 standard deviations lower** (1.3 to 0.36 lower) |  | 490 (6 studies) | ⊕⊕⊕⊝ **moderate**^2^ | SMD -0.83 (-1.3 to -0.36) |
| **Pain on movement - Pain (4w)** |  | The mean pain on movement - pain (4w) in the intervention groups was **0.98 standard deviations lower** (1.33 to 0.62 lower) |  | 137 (3 studies) | ⊕⊕⊕⊝ **moderate**^1^ | SMD -0.98 (-1.33 to -0.62) |
| **Pain on movement - Pain (6w)** |  | The mean pain on movement - pain (6w) in the intervention groups was **0.99 standard deviations lower** (1.84 to 0.13 lower) |  | 524 (5 studies) | ⊕⊕⊕⊝ **moderate**^2^ | SMD -0.99 (-1.84 to -0.13) |
| **Pain on movement - Pain (8w)** |  | The mean pain on movement - pain (8w) in the intervention groups was **1.23 standard deviations lower** (1.72 to 0.73 lower) |  | 77 (2 studies) | ⊕⊕⊕⊝ **moderate**^1^ | SMD -1.23 (-1.72 to -0.73) |
| **Pain on movement - Pain (12w)** |  | The mean pain on movement - pain (12w) in the intervention groups was **0.59 standard deviations lower** (1.28 lower to 0.1 higher) |  | 358 (3 studies) | ⊕⊕⊝⊝ **low**^1,2^ | SMD -0.59 (-1.28 to 0.1) |
| **Pain on movement - Pain (6m)** |  | The mean pain on movement - pain (6m) in the intervention groups was **0.13 standard deviations higher** (0.18 lower to 0.43 higher) |  | 350 (2 studies) | ⊕⊕⊝⊝ **low**^1,2^ | SMD 0.13 (-0.18 to 0.43) |
| **Pain on movement - Pain (12m)** |  | The mean pain on movement - pain (12m) in the intervention groups was **0.06 standard deviations higher** (0.15 lower to 0.27 higher) |  | 350 (2 studies) | ⊕⊕⊕⊝ **moderate**^1^ | SMD 0.06 (-0.15 to 0.27) |
| **Function - Function (1w)** |  | The mean function - function (1w) in the intervention groups was **1.79 standard deviations lower** (2.56 to 1.01 lower) |  | 105 (2 studies) | ⊕⊕⊝⊝ **low**^1,2^ | SMD -1.79 (-2.56 to -1.01) |
| **Function - Function (2w)** |  | The mean function - function (2w) in the intervention groups was **1.44 standard deviations lower** (1.89 to 0.99 lower) |  | 105 (2 studies) | ⊕⊕⊕⊝ **moderate**^1^ | SMD -1.44 (-1.89 to -0.99) |
| **Function - Function (4w)** |  | The mean function - function (4w) in the intervention groups was **1.54 standard deviations lower** (2.62 to 0.46 lower) |  | 137 (3 studies) | ⊕⊕⊝⊝ **low**^1,2^ | SMD -1.54 (-2.62 to -0.46) |
| **Function - Function (8w)** |  | The mean function - function (8w) in the intervention groups was **1.13 standard deviations lower** (1.77 to 0.49 lower) |  | 77 (2 studies) | ⊕⊕⊝⊝ **low**^1,2^ | SMD -1.13 (-1.77 to -0.49) |
| **ROM of knee - ROM of knee (1w)** |  | The mean rom of knee - rom of knee (1w) in the intervention groups was **2.85 higher** (0.78 lower to 6.48 higher) |  | 139 (2 studies) | ⊕⊕⊕⊝ **moderate**^1^ |  |
| **ROM of knee - ROM of knee (6w)** |  | The mean rom of knee - rom of knee (6w) in the intervention groups was **4.25 higher** (2.2 to 6.31 higher) |  | 278 (2 studies) | ⊕⊕⊕⊝ **moderate**^1^ |  |
| **ROM of knee - ROM of knee (12w)** |  | The mean rom of knee - rom of knee (12w) in the intervention groups was **1.39 higher** (0.43 lower to 3.21 higher) |  | 378 (3 studies) | ⊕⊕⊕⊝ **moderate**^1^ |  |
| **Analgesics consumption - PCA consumption** |  | The mean analgesics consumption - pca consumption in the intervention groups was **8.2 lower** (19.93 lower to 3.54 higher) |  | 405 (4 studies) | ⊕⊕⊕⊝ **moderate**^2^ |  |
| **Analgesics consumption - Opioids consumption (24h)** |  | The mean analgesics consumption - opioids consumption (24h) in the intervention groups was **7.26 lower** (14.34 to 0.18 lower) |  | 366 (3 studies) | ⊕⊕⊝⊝ **low**^1,2^ |  |
| **Analgesics consumption - Opioids consumption (≥7d)** |  | The mean analgesics consumption - opioids consumption (≥7d) in the intervention groups was **10.81 lower** (22.35 lower to 0.73 higher) |  | 366 (3 studies) | ⊕⊕⊝⊝ **low**^1,2^ |  |
| **Depression** |  | The mean depression in the intervention groups was **2.74 lower** (3.91 to 1.56 lower) |  | 157 (3 studies) | ⊕⊕⊕⊝ **moderate**^1^ |  |
| **Mental health** |  | The mean mental health in the intervention groups was **11.66 higher** (8.44 to 14.89 higher) |  | 112 (2 studies) | ⊕⊕⊕⊝ **moderate**^1^ |  |
| *The basis for the **assumed risk** (e.g. the median control group risk across studies) is provided in footnotes. The **corresponding risk** (and its 95% confidence interval) is based on the assumed risk in the comparison group and the **relative effect** of the intervention (and its 95% CI).  **CI:** Confidence interval; | | | | | | |
| GRADE Working Group grades of evidence **High quality:** Further research is very unlikely to change our confidence in the estimate of effect.  **Moderate quality:** Further research is likely to have an important impact on our confidence in the estimate of effect and may change the estimate. **Low quality:** Further research is very likely to have an important impact on our confidence in the estimate of effect and is likely to change the estimate. **Very low quality:** We are very uncertain about the estimate. | | | | | | |
| ^1^ imprecision of results due to sparse data (total population size was less than 400) ^2^ substantial heterogeneity | | | | | | |

**Figure F1** PRISMA flowchart of study selection

**
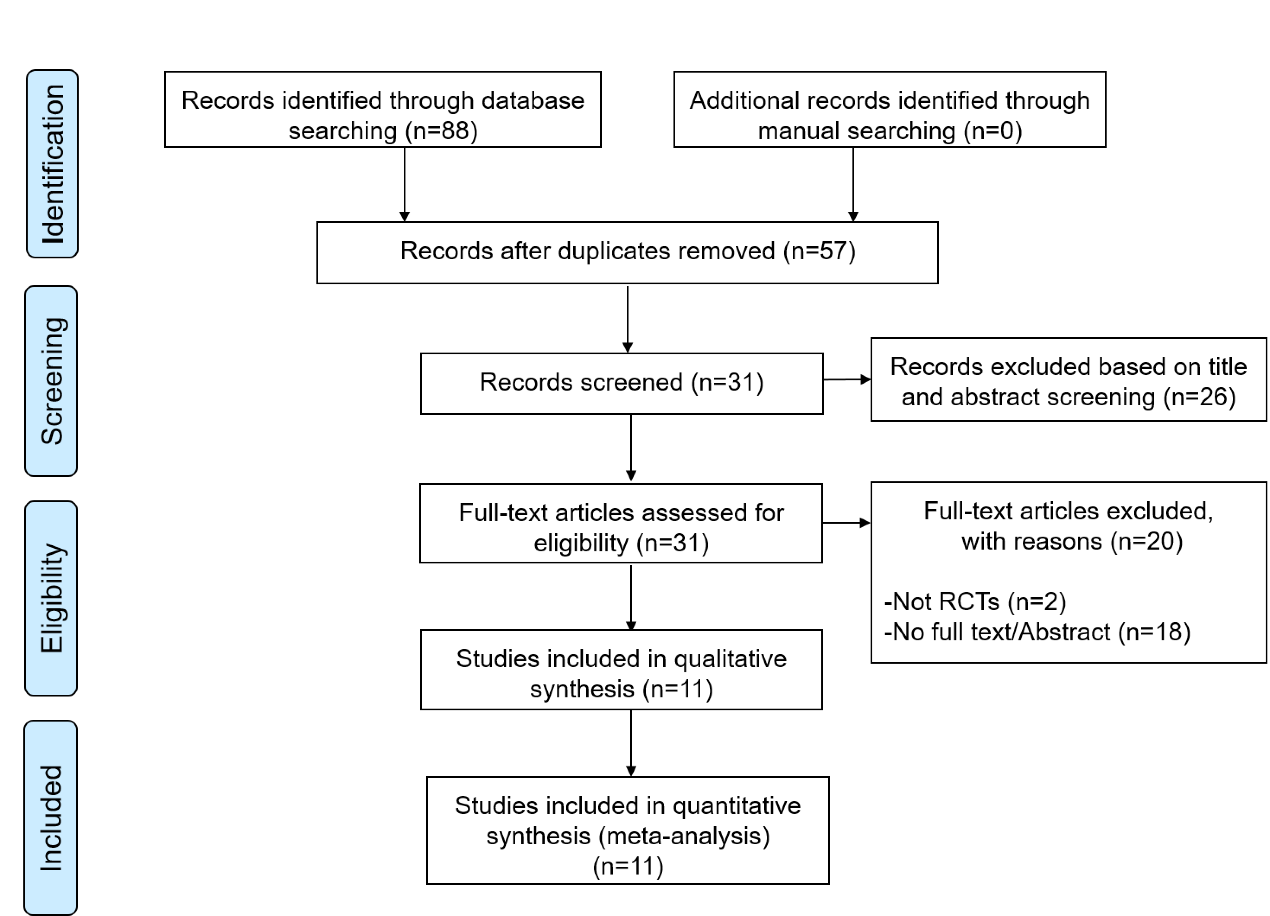
**

**Figure F2 Trial sequential analysis for ROM of knee at 1 week**


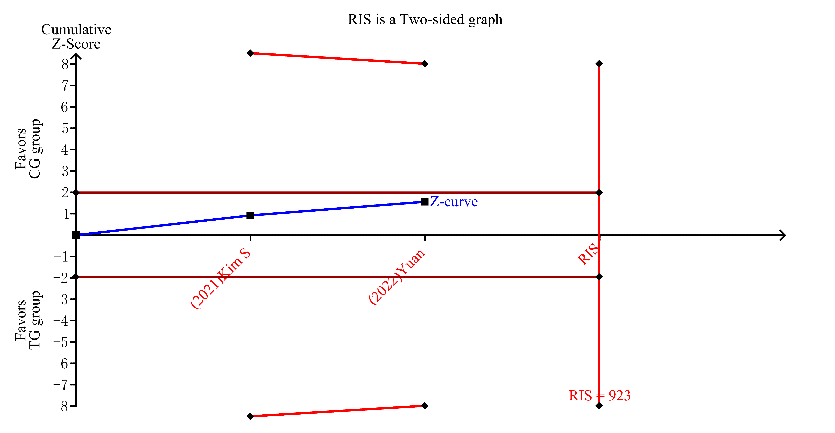


**Figure F3 Trial sequential analysis for ROM of knee at 6 weeks**

**
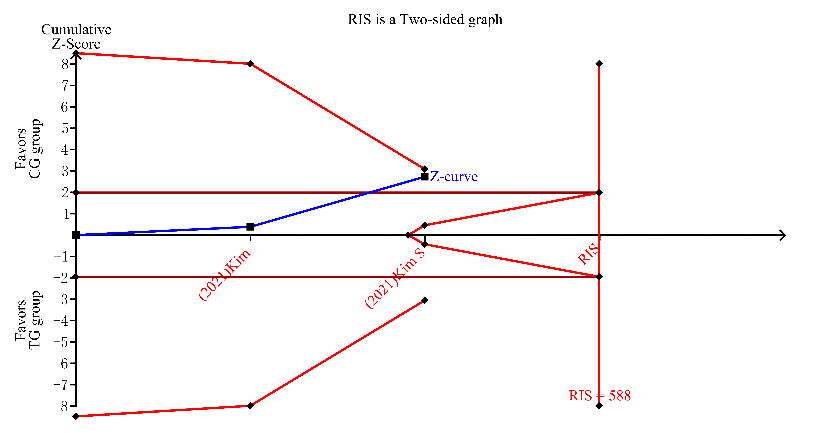
**

**Figure F4 Trial sequential analysis for ROM of knee at 12 weeks**

**
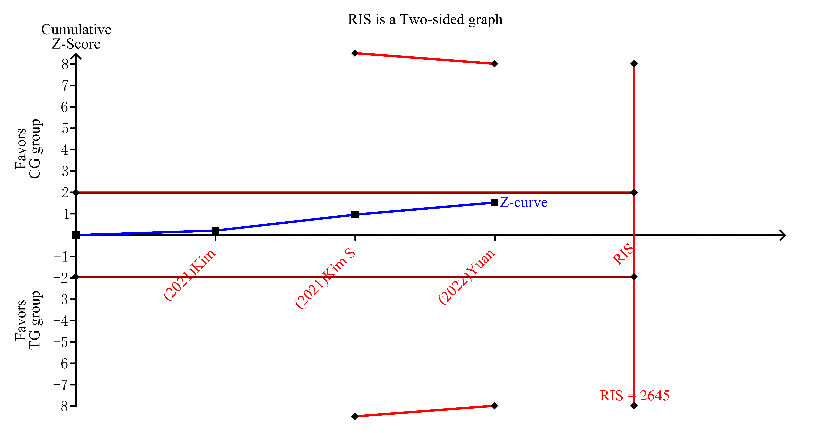
**

**Figure F5 Trial sequential analysis for patient-controlled analgesia consumption**

**
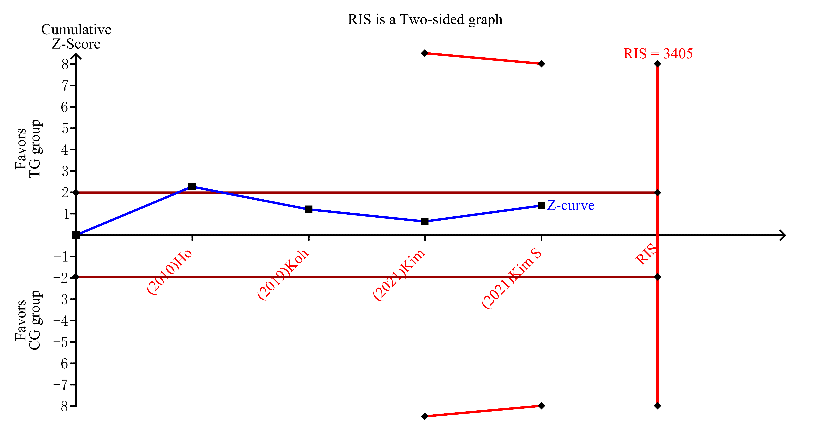
**

**Figure F6 Trial sequential analysis for opioids consumption at 24 h**

**
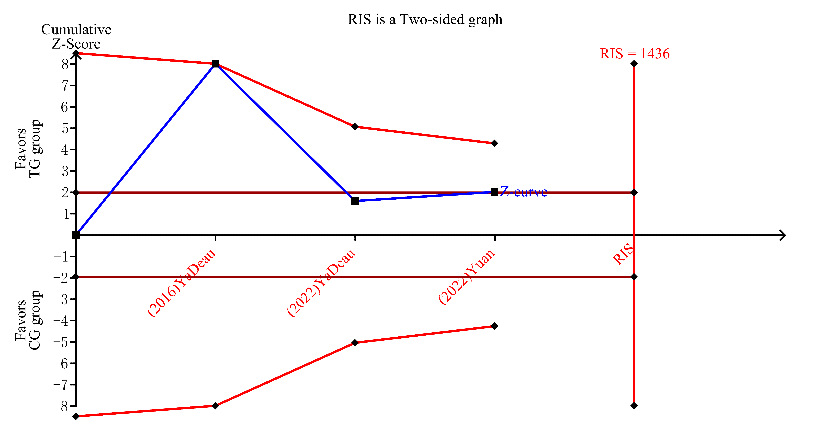
**

**Figure F7 Trial sequential analysis for opioids consumption more than 7 days**

**
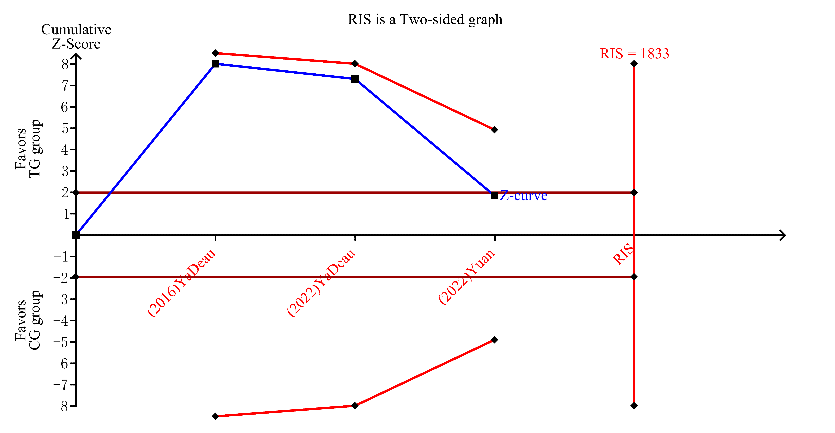
**

**Figure F8 Trial sequential analysis for depression**

**
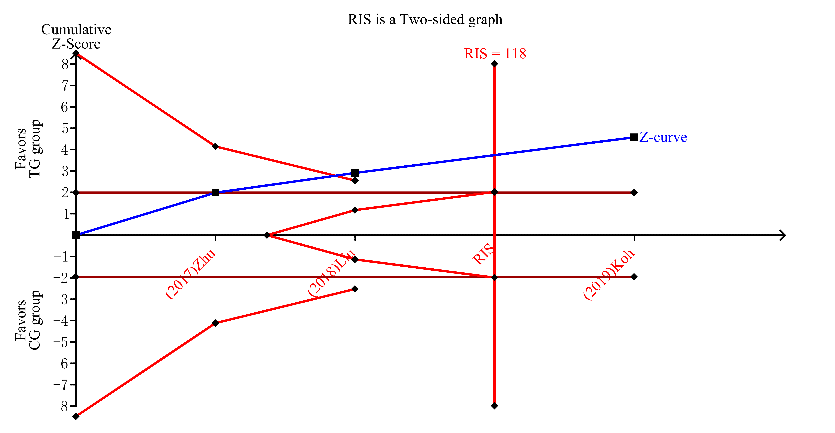
**

**Figure F9 Trial sequential analysis for mental health**


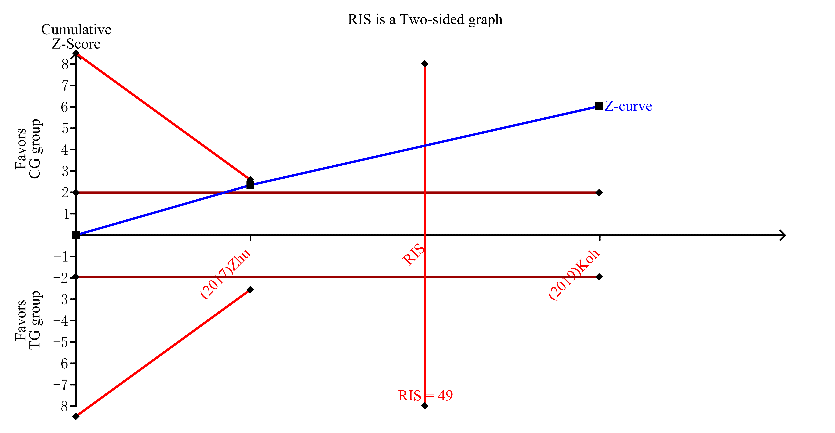


**Table S5** AMSTAR checklist


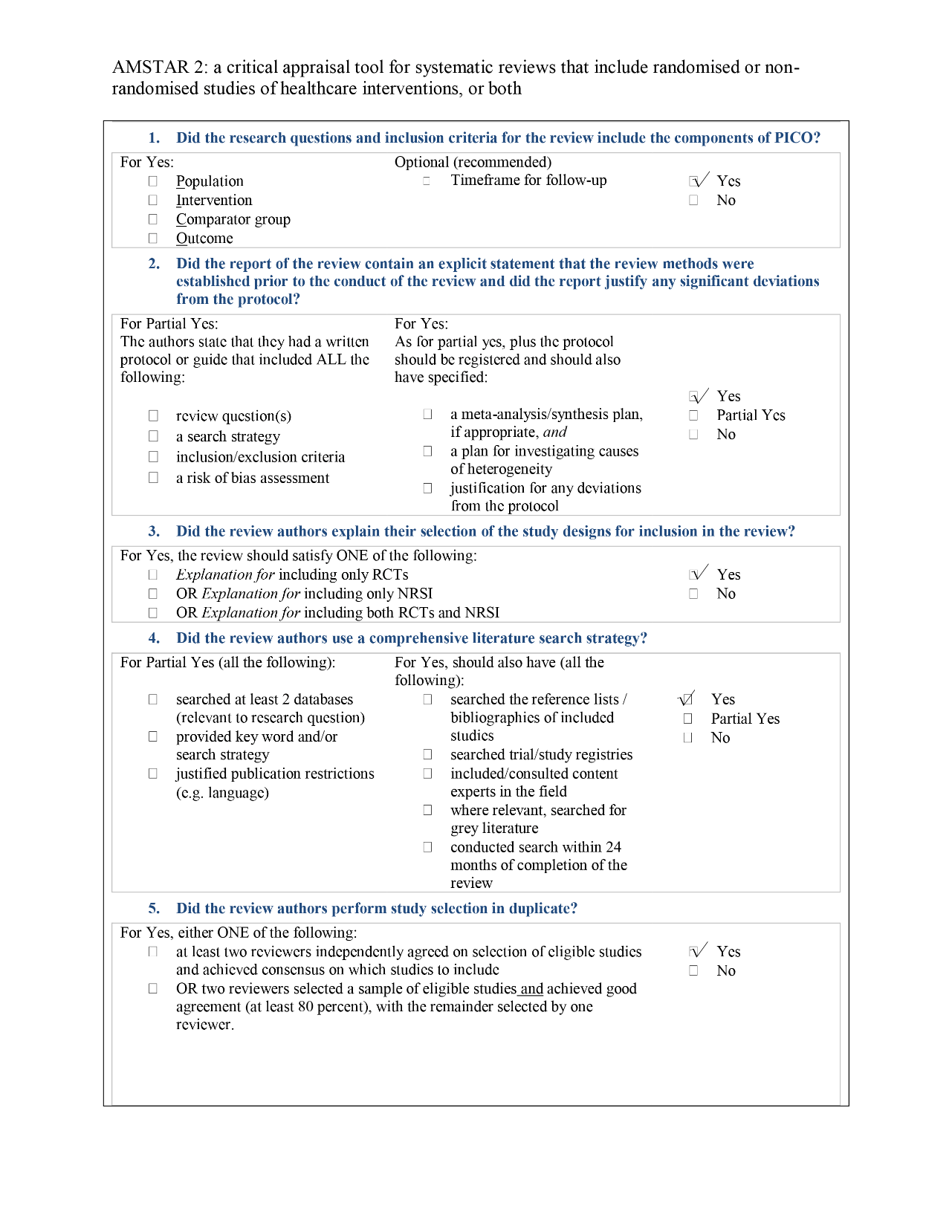


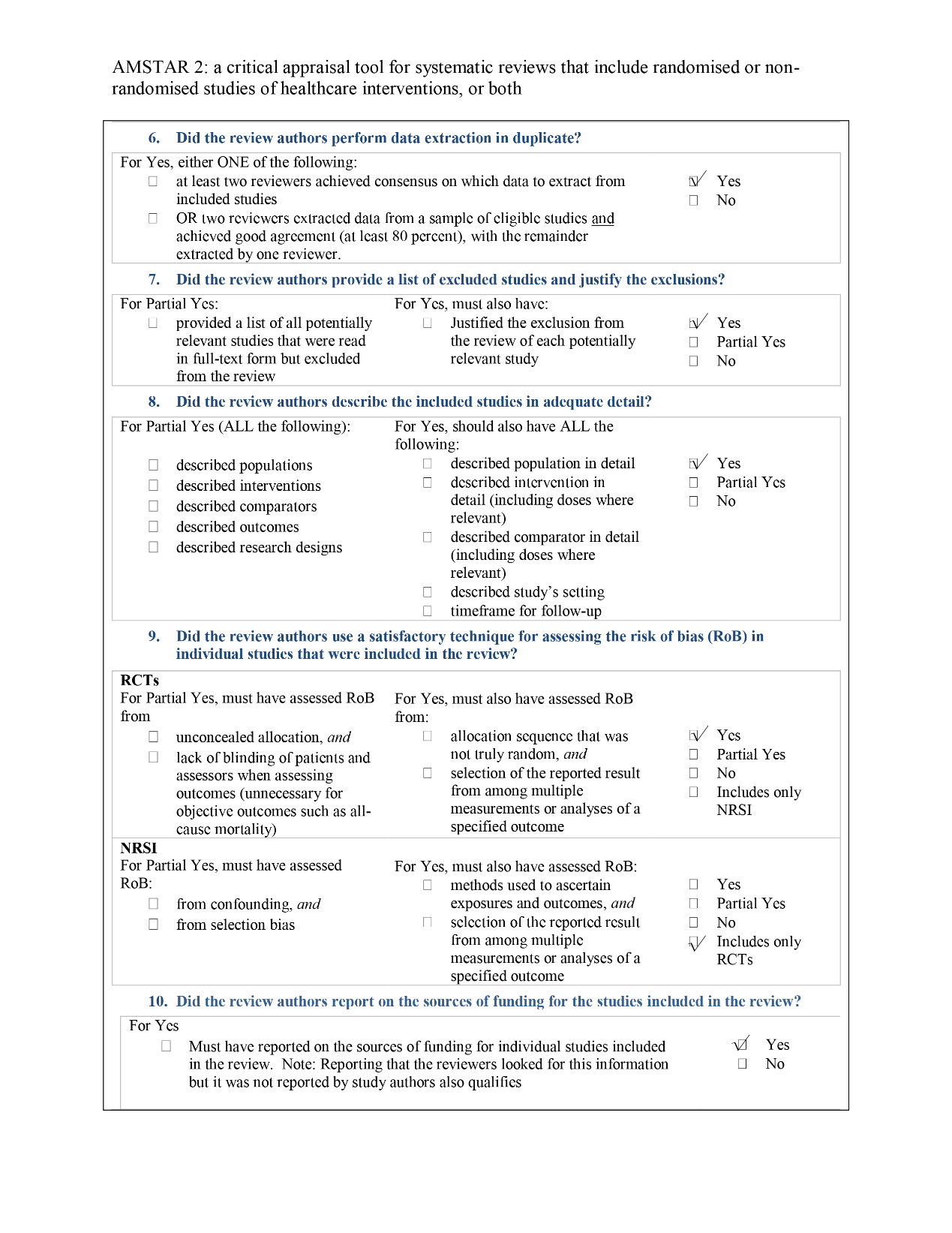


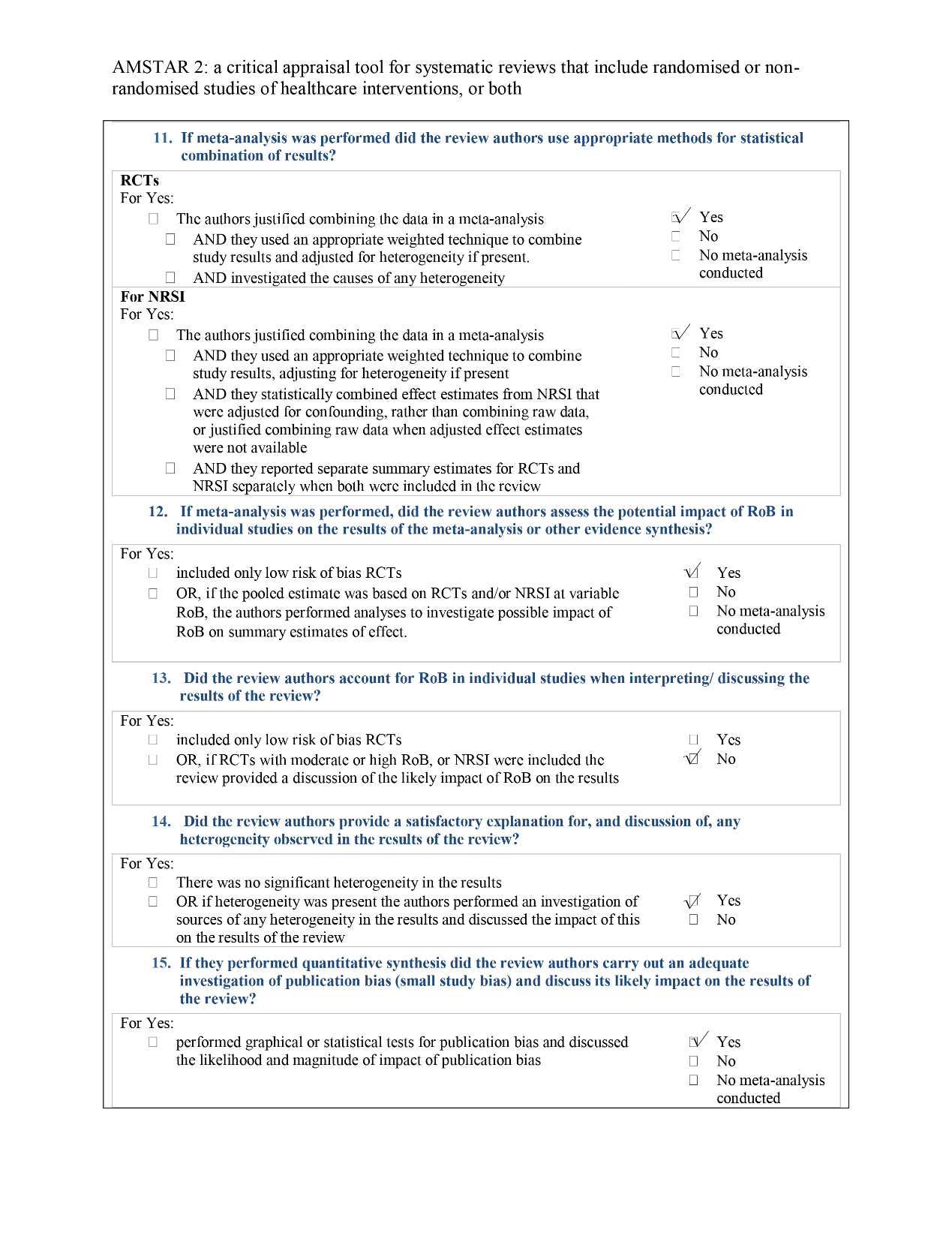


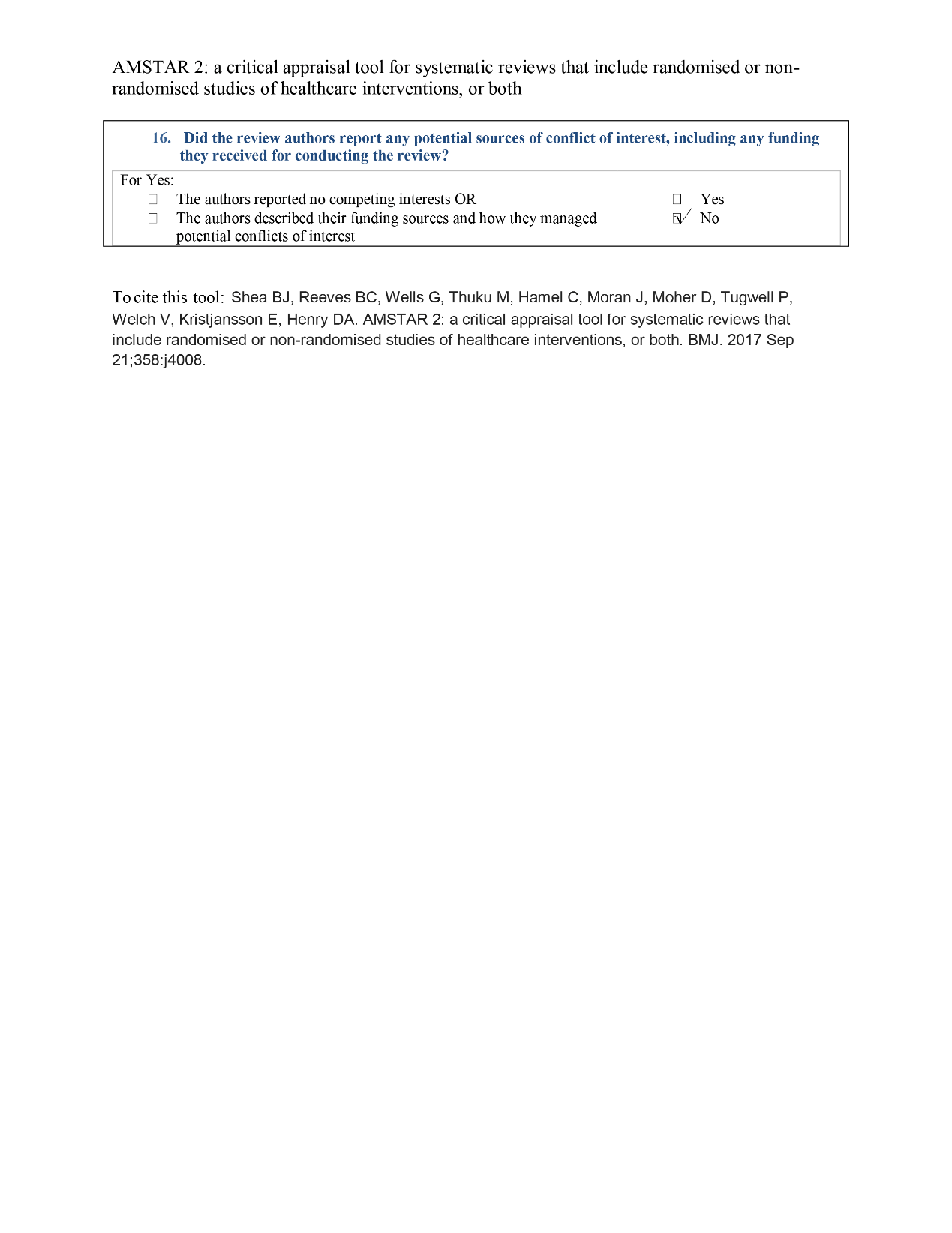


**Table S6** PRISMA 2020 checklist


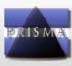
**PRISMA 2020 Checklist**

| **Section and Topic** | **Item #** | **Checklist item** | **Location where item is reported** |
| --- | --- | --- | --- |
| **TITLE** | | |  |
| Title | 1 | Identify the report as a systematic review. | 1 |
| **ABSTRACT** | | |  |
| Abstract | 2 | See the PRISMA 2020 for Abstracts checklist. | 1 |
| **INTRODUCTION** | | |  |
| Rationale | 3 | Describe the rationale for the review in the context of existing knowledge. | 1 2 |
| Objectives | 4 | Provide an explicit statement of the objective(s) or question(s) the review addresses. | 1 2 |
| **METHODS** | | |  |
| Eligibility criteria | 5 | Specify the inclusion and exclusion criteria for the review and how studies were grouped for the syntheses. | 3 |
| Information sources | 6 | Specify all databases, registers, websites, organisations, reference lists and other sources searched or consulted to identify studies. Specify the date when each source was last searched or consulted. | 3 |
| Search strategy | 7 | Present the full search strategies for all databases, registers and websites, including any filters and limits used. | 3 |
| Selection process | 8 | Specify the methods used to decide whether a study met the inclusion criteria of the review, including how many reviewers screened each record and each report retrieved, whether they worked independently, and if applicable, details of automation tools used in the process. | 3 |
| Data collection process | 9 | Specify the methods used to collect data from reports, including how many reviewers collected data from each report, whether they worked independently, any processes for obtaining or confirming data from study investigators, and if applicable, details of automation tools used in the process. | 3 |
| Data items | 10a | List and define all outcomes for which data were sought. Specify whether all results that were compatible with each outcome domain in each study were sought (e.g. for all measures, time points, analyses), and if not, the methods used to decide which results to collect. | 5 |
|  | 10b | List and define all other variables for which data were sought (e.g. participant and intervention characteristics, funding sources). Describe any assumptions made about any missing or unclear information. | 5 |
| Study risk of bias assessment | 11 | Specify the methods used to assess risk of bias in the included studies, including details of the tool(s) used, how many reviewers assessed each study and whether they worked independently, and if applicable, details of automation tools used in the process. | 3 4 |
| Effect measures | 12 | Specify for each outcome the effect measure(s) (e.g. risk ratio, mean difference) used in the synthesis or presentation of results. | 4 |
| Synthesis methods | 13a | Describe the processes used to decide which studies were eligible for each synthesis (e.g. tabulating the study intervention characteristics and comparing against the planned groups for each synthesis (item #5)). | 4 |
|  | 13b | Describe any methods required to prepare the data for presentation or synthesis, such as handling of missing summary statistics, or data conversions. | 4 |
|  | 13c | Describe any methods used to tabulate or visually display results of individual studies and syntheses. | 4 |
|  | 13d | Describe any methods used to synthesize results and provide a rationale for the choice(s). If meta-analysis was performed, describe the model(s), method(s) to identify the presence and extent of statistical heterogeneity, and software package(s) used. | 4 |
|  | 13e | Describe any methods used to explore possible causes of heterogeneity among study results (e.g. subgroup analysis, meta-regression). | 4 |
|  | 13f | Describe any sensitivity analyses conducted to assess robustness of the synthesized results. | 4 |
| Reporting bias assessment | 14 | Describe any methods used to assess risk of bias due to missing results in a synthesis (arising from reporting biases). | 4 |
| Certainty assessment | 15 | Describe any methods used to assess certainty (or confidence) in the body of evidence for an outcome. | 4 |
| **RESULTS** | | |  |
| Study selection | 16a | Describe the results of the search and selection process, from the number of records identified in the search to the number of studies included in the review, ideally using a flow diagram. | 4 |
|  | 16b | Cite studies that might appear to meet the inclusion criteria, but which were excluded, and explain why they were excluded. | 4 |
| Study characteristics | 17 | Cite each included study and present its characteristics. | 4 5 6 |
| Risk of bias in studies | 18 | Present assessments of risk of bias for each included study. | 7 |
| Results of individual studies | 19 | For all outcomes, present, for each study: (a) summary statistics for each group (where appropriate) and (b) an effect estimate and its precision (e.g. confidence/credible interval), ideally using structured tables or plots. | 5 6 |
| Results of syntheses | 20a | For each synthesis, briefly summarise the characteristics and risk of bias among contributing studies. | 7 8 9 |
|  | 20b | Present results of all statistical syntheses conducted. If meta-analysis was done, present for each the summary estimate and its precision (e.g. confidence/credible interval) and measures of statistical heterogeneity. If comparing groups, describe the direction of the effect. | 7 8 9 |
|  | 20c | Present results of all investigations of possible causes of heterogeneity among study results. | 7 8 9 |
|  | 20d | Present results of all sensitivity analyses conducted to assess the robustness of the synthesized results. | 7 8 9 |
| Reporting biases | 21 | Present assessments of risk of bias due to missing results (arising from reporting biases) for each synthesis assessed. | 10 11 |
| Certainty of evidence | 22 | Present assessments of certainty (or confidence) in the body of evidence for each outcome assessed. | 10 11 |
| **DISCUSSION** | | |  |
| Discussion | 23a | Provide a general interpretation of the results in the context of other evidence. | 11 |
|  | 23b | Discuss any limitations of the evidence included in the review. | 11 12 13 |
|  | 23c | Discuss any limitations of the review processes used. | 12 13 |
|  | 23d | Discuss implications of the results for practice, policy, and future research. | 11 12 |
| **OTHER INFORMATION** | | |  |
| Registration and protocol | 24a | Provide registration information for the review, including register name and registration number, or state that the review was not registered. | 2 |
|  | 24b | Indicate where the review protocol can be accessed, or state that a protocol was not prepared. | 2 |
|  | 24c | Describe and explain any amendments to information provided at registration or in the protocol. | 2 |
| Support | 25 | Describe sources of financial or non-financial support for the review, and the role of the funders or sponsors in the review. | 13 |
| Competing interests | 26 | Declare any competing interests of review authors. | 13 |
| Availability of data, code and other materials | 27 | Report which of the following are publicly available and where they can be found: template data collection forms; data extracted from included studies; data used for all analyses; analytic code; any other materials used in the review. | 3 4 9 11 |
